# Estrogen-Dependent Gene Expression in the Mouse Ovary

**DOI:** 10.1371/journal.pone.0014672

**Published:** 2011-02-09

**Authors:** Seng H. Liew, Mai A. Sarraj, Ann E. Drummond, Jock K. Findlay

**Affiliations:** 1 Prince Henry's Institute of Medical Research, Clayton, Melbourne, Australia; 2 Department of Obstetrics and Gynaecology, Monash University, Melbourne, Australia; Massachusetts General Hospital, United States of America

## Abstract

Estrogen (E) plays a pivotal role in regulating the female reproductive system, particularly the ovary. However, the number and type of ovarian genes influenced by estrogen remain to be fully elucidated. In this study, we have utilized wild-type (WT) and aromatase knockout (ArKO; estrogen free) mouse ovaries as an *in vivo* model to profile estrogen dependent genes. RNA from each individual ovary (n = 3) was analyzed by a microarray-based screen using Illumina Sentrix Mouse WG-6 BeadChip (45,281 transcripts). Comparative analysis (GeneSpring) showed differential expression profiles of 450 genes influenced by E, with 291 genes up-regulated and 159 down-regulated by 2-fold or greater in the ArKO ovary compared to WT. Genes previously reported to be E regulated in ArKO ovaries were confirmed, in addition to novel genes not previously reported to be expressed or regulated by E in the ovary. Of genes involved in 5 diverse functional processes (hormonal processes, reproduction, sex differentiation and determination, apoptosis and cellular processes) 78 had estrogen-responsive elements (ERE). These analyses define the transcriptome regulated by E in the mouse ovary. Further analysis and investigation will increase our knowledge pertaining to how E influences follicular development and other ovarian functions.

## Introduction

The importance of estrogen (E) in female reproductive endocrinology and in ovarian function has been well documented [1]. Estrogen signalling is primarily transduced by estrogen receptors (ER) α and β [2]. ER are members of a conserved superfamily of ligand activated transcription factors. The effects of E on ER are exerted through a complex array of convergent and divergent signaling pathways that mediate genomic events involved in regulation of mitogenesis, differentiation and apoptosis [3], [4]. The interaction of E and ER with specific DNA sequences called estrogen responsive elements (EREs), constitutes a primary genomic signaling pathway [3], [4]. The ERE-bound ER recruits an ensemble of co-factors responsible for the alteration of local chromatin structure and interaction with the basal transcription machinery.

Much effort has been invested in developing techniques to identify genes of interest, such as, Northern blot, semi-quantitative RT-PCR and serial analysis of gene expression (SAGE). Several candidate genes, such as *cyclin D2* [5], growth and differentiation factor 9 (*GDF-9*) [5], forkhead box transcription factor (*Foxo1*) [6], inhibinα and inhibinβB [7] were shown to be regulated by E using these approaches. However, many downstream gene targets of E were unlikely to have been discovered using this approach. The aromatase knockout (ArKO) female mouse is deficient in aromatase activity postnatally and therefore is an excellent model to allow us to define E-dependent ovarian genes in adulthood. Using RT-PCR we identified genes usually associated with male reproduction such as *Sox9*, *DAX1*, liver homolog-1 (*Lrh-1*) and Mullerian-inhibiting substance [8] as being significantly increased [8] in the ArKO ovary. Steroidogenic enzymes such as *17α-OHase*, *17ß-Hsd1*, and *17β-Hsd3* mRNA's were also increased and the patterns of expression of the steroidogenic enzymes responsible for androgen biosynthesis in the ArKO ovaries correlated with increased serum testosterone levels in ArKO females [8].

The development of microarray technology now enables the simultaneous measurement of thousands of gene transcripts in a biological sample [9]. Therefore, in order to identify E-dependent genes in the ovary, this study utilized a microarray approach profiling wildtype (WT) and ArKO ovaries. The study aimed to 1) confirm the preliminary observations on estrogen dependent genes, 2) provide novel information about genes that can be used to unravel the mechanism of E in maintaining the female gonad, and 3) provide new insights into the regulation of ovarian follicular development.

## Materials and Methods

### Animals

Wild-type (WT) and ArKO mice on a J129/C57B6 background were maintained under specific pathogen-free (SPF) conditions, on a 12L∶12D regimen and fed *ad libitum* a soy free mouse chow (Glen Forrest Stockfeeders, Western Australia). All animal procedures were approved by a Monash University Animal Ethics Committee (Project number: MMCB2002/37) and were carried out in accordance with the Australian Code of Practice for the Care and Use of Animals for Scientific Purposes.

### Experimental design

**Figure 1:**
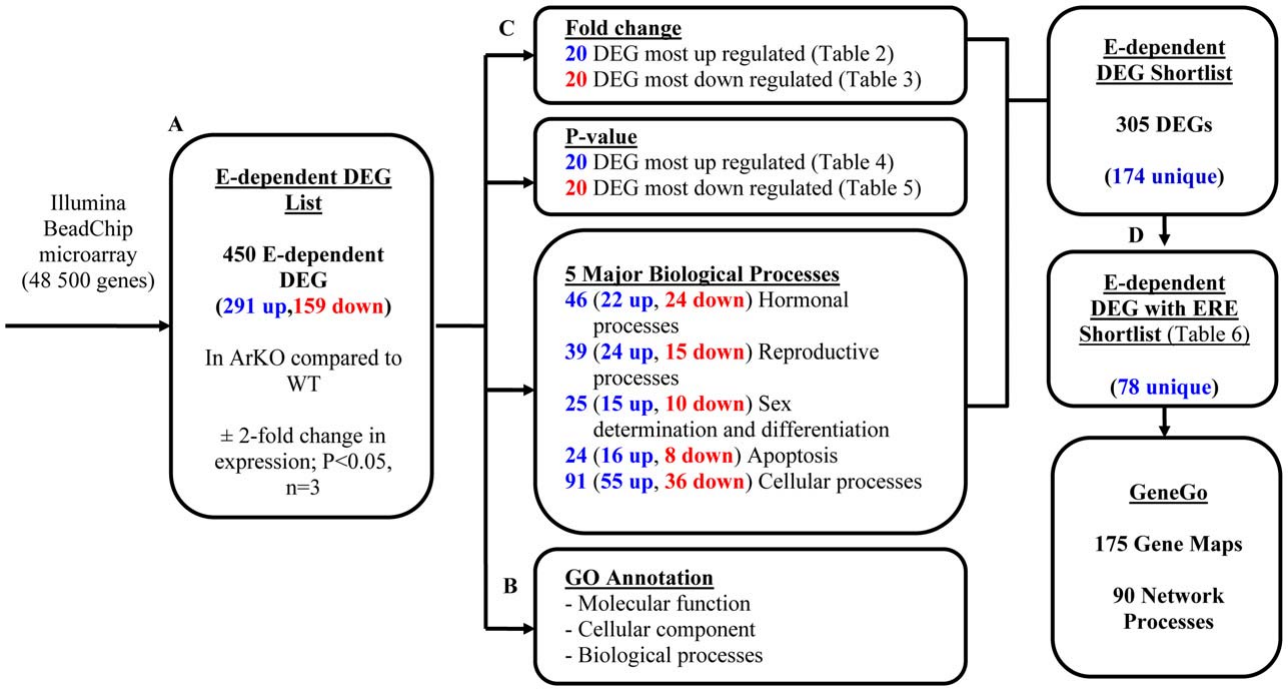
Flowchart of microarray data ranking and analysis. **A**) 450 E-dependent DEG list were identified by microarray in ovaries of ArKO vs WT as having ±2-fold change expression with p-value<0.05. **B**) The E-dependent DEG list was annotated using Gene Ontology and analysed for molecular function, cellular component and biological processeses. **C**) E-dependent DEG from the list were ranked based upon fold change of up/down regulation (Fold change); significance of the change (P-value); 5 major GeneGo biological processes genes: Hormonal processes, Reproductive processes, Sex determination and differentiation, Apoptosis and, Cellular processes. **D**) Genes that possess EREs. Genes identified from the list using these methods were compiled to form an E-dependent DEG with ERE Shortlist which was used for downstream analysis of gene networks and pathways affected by E using GeneGo pathway analysis. The E-dependent DEG list can be found in Table S1.

The experimental approach is summarized in Figure 1. WT and ArKO mice, 16 weeks of age (n = 3/grp) were killed by CO_2_ asphyxiation. The abdomen was opened and both ovaries from each animal were collected and snap frozen in liquid nitrogen. RNA was extracted from individual ovaries using a phenol-chloroform-based method (Ultraspec; Fisher Biotech, Subiaco, Western Australia, Australia). Quantification of RNA concentration and purity was measured using the NanoDrop spectrophotometer (Thermo Scientific). The quality of the mouse RNA was ascertained using the Agilent Bioanalyser 2100 using the NanoChip protocol. A total of 500ng RNA was amplified and labeled using the Illumina TotalPrep RNA Amplification kit (Ambion) as per the manufacturer's instructions. A total of 1.5ug of labelled cRNA was then prepared for hybridisation to the Sentrix Mouse-6 Expression Beadchip (v1.1) by preparing a probe cocktail (cRNA @ 0.05ug/ul) that includes GEX-HYB Hybridisation Buffer (supplied with the beadchip). A total hybridisation volume of 30ul is prepared for each sample and 30ul loaded into a single array on the Sentrix Mouse-6 Expression Beadchip (v1.1). The Sentrix Mouse-6 Expression Beadchip (v1.1) allows for six samples and targets 48,500 unique well-documented RefSeq transcripts. A total of 6 different labelled samples can be loaded into 6 individual arrays per beadchip. The chip is hybridised at 58°C for 16 hours in an oven with a rocking platform. After hybridisation, the chip is washed using protocols outlined in the Illumina manual. Upon completion of the washing, the chips are then coupled with Cy3 and scanned in the Illumina BeadArray Reader. The scanner operating software, BeadStudio, converts the signal on the array into a text file for analysis. All data is MIAME compliant and the raw data has been deposited in a MIAME compliant database ArrayExpress (ArrayExpress accession: E-MEXP-2824). The Illumina microarray was performed by the Australian Genome Research Facility (AGRF) Melbourne, Australia.

### Statistical analysis for microarray data

Prior to differential gene expression analysis, quality control diagnostic measurements were run on the raw data generated from the Illumina chips. 6 Sentrix Mouse-6 Expression Beadchip (v1.1) samples were normalised using lumi package (part of bioconductor) in R statistical software and were subsequently imported into GeneSpring GX software for further analysis. The significant E-dependent differentially expressed genes (DEG) were obtained from comparing KO samples to WT samples and were defined by a fold difference of 2.0 and a P-value cut-off of 0.05 (derived from ANOVA). Genes that met these parameters were included in the E-dependent DEG list and colour coded with blue representing down-regulation and red representing up-regulation of transcript when comparing KO samples to WT samples. The data normalisation and statistical analysis to generate E-dependent DEG list was performed by the AGRF Melbourne, Australia.

### Bioinformatic analysis and downstream annotation

E-dependent DEG were ranked according to the methods described in the results section. Specific methods for Gene Ontology, GeneGo pathways analysis and Dragon estrogen response element (ERE) Finder version 6 are detailed below.

#### 1. Gene Ontology (GO) [6] Analysis

E-dependent DEG lists were annotated according to the GO database (http://www.geneontology.org) using ontology categories for Molecular Function, Cellular Component and Biological Processes. The representation of E-dependent DEG within each ontology category was measured and statistical significance of the overlap was done using a hypergeometric p-value without multiple testing corrections, to determine the likelihood of coincidental overlap with ontology groups. A p-value lower than 0.05 indicates over representation of genes from the E-dependent DEG list within that particular category, suggestive of a functional effect. Annotation of the E-dependent DEG lists using GO was conducted by the AGRF Melbourne, Australia.

#### 2. Dragon ERE Locator version 6

Nucleotide search from National Center for Biotechnology Information (http://www.ncbi.nlm.nih.gov/nucleotide/) was used to retrieve the sequence information of the differentially expressed genes within the selected Biological Processes categories in the FASTA format. These sequences were then examined for the presence of ERE binding sites using the latest Dragon ERE Finder version 6.0 (http://apps.sanbi.ac.za/ere/index.php) [10] based upon information present in the transcriptional regulation, from patterns to profiles (TRANSFAC) database [11]. The detection algorithm was tested on several large datasets and achieved a sensitivity of 83% [10]. An ERE is defined as a site which contains the 17 basepairs consensus sequence with its flanking 20 nucleotides on either side against the promoter sequences of Eukaryotic Promoter Database (EPD) [10], [12], [13].

#### 3. GeneGo pathway analysis

The E-dependent DEG list containing the 450 unique E-dependent DEG, complete with Illumina transcript identifiers, were uploaded from a Microsoft Excel spreadsheet onto Metacore 5.0 software (GeneGo pathways analysis) (http://www.genego.com). GeneGo recognizes the Illumina identifiers and maps the E-dependent DEG to the MetaCore™ data analysis suite, generating maps to describe common pathways or molecular connections between E-dependent DEG on the list. Graphical representations of the molecular relationships between genes were generated using the GeneGo pathway analysis, based upon processes showing significant (P<0.05) association.

## Results

### E-dependent Differentially expressed genes (DEG) in ArKO compared to WTs

Microarray analysis of whole genome expression in ArKO ovary compared to WT ovary identified 450 differentially expressed transcripts with a ±2-fold expression difference that was significant (p<0.05 according to t-tests, n = 3) (Table 1). This list is referred to as the E-dependent DEG list and can be found in full in the supplementary data. The 450 E-dependent DEGs represent ∼0.94% of the total number of transcripts present on the Illumina BeadChip. Of these 450 E-dependent DEG, 291 were up regulated (within a range of 2 to 47-fold change), and 159 DEG were down regulated (range between −2 to −15.7-fold change) in the absence of E.

**Table 1:** E-dependent DEG in ArKO ovary compared to WT ovary.

Fold Change	Differential Expressed Genes (DEG)		
	Up	Down	total
47∼2.0	291	-	291
−2.0∼−15.7	-	159	159
Total			450

Up; up regulated within designated fold range. Down; down regulated within designated fold range. All E-dependent DEG were significantly differentially expressed (p<0.05) (n = 3).

### GO annotation of the E-dependent DEG list

We hypothesised that some of the E-dependent DEG would be involved in downstream molecular pathways important for maintaining the female gonad. In order to identify which types of global cellular processes or specific molecular functions were responsive to E, the E-dependent DEG list was annotated using the GO [6] database. Each of the 450 genes was assigned to Molecular Function, Cellular Component and Biological Process categories as designated by the GO database. GO categories significantly over-represented in the E-dependent DEG list are those that match a greater number of the E-dependent DEG than would be expected by chance (based upon comparison to the total number of genes in the genome assigned to the category).

**Figure 2:**
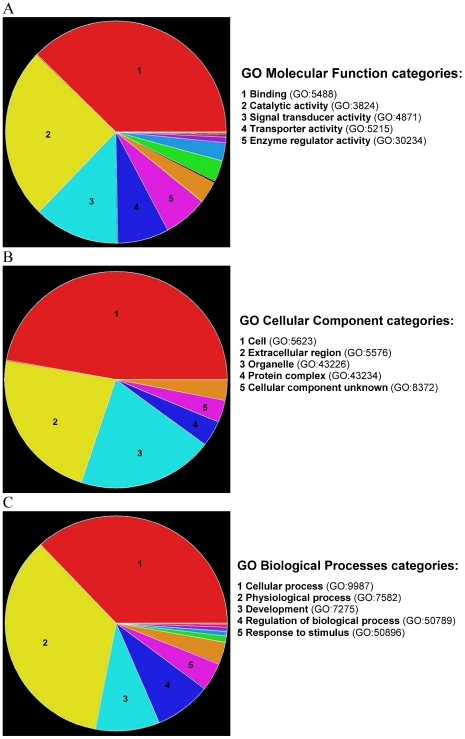
Gene Ontology (GO) annotation E-dependent DEG list with Molecular Function, Cellular Component and Biological Processes categories. **A**) Pie chart shows the distribution of the 388 E-dependent DEG that were matched to a Molecular Function using GO [6]. **B**) Pie chart shows the distribution of the 319 E-dependent DEG that were matched to a Cellular Component using GO [6]. **C**) Pie chart shows the distribution of the 302 E-dependent DEG that were matched to a Biological Processes using GO [6].

Figure 2A summarises the annotation of the E-dependent DEG list with a Molecular Function using GO. 388 of the 450 genes were matched to categories and the distribution of these 388 E-dependent DEG into GO Molecular Function categories. The five GO categories with the greatest proportion of E-dependent DEG were Binding, Catalytic activity, Signal Transducer activity, Transporter activity and Enzyme regulator activity.

Figure 2B summarises the annotation of the E-dependent DEG list with a Cellular Component using GO. 319 of the 450 genes were matched to categories and the distribution of these 319 E-dependent DEG into GO Cellular Component categories. The five GO categories with the greatest proportion of E-dependent DEG were Cell, Extracellular region, Organelle, Protein complex and Cellular component unknown.

Figure 2C summarises the annotation of the E-dependent DEG list to GO Biological Processes. 302 of the 450 genes were matched to categories and the distribution of these 302 E-dependent DEG into GO Biological Process categories. The GO categories with the greatest proportion of E-dependent DEGs were Cellular process, Physiological process, Development, Regulation of Biological Process and Response to Stimulus.

### Ranking of genes on the E-dependent DEG list to create an E-dependent DEG with ERE Shortlist

As described in Table 1, 450 E-dependent DEG were identified in ArKO ovaries that showed expression ±2-fold compared to WT, with a significance of p<0.05 (E-dependent DEG list, Table S1). In order to narrow the E-dependent DEG list to those most likely to represent direct biological E target genes, four approaches were used to create a ranked shortlist of E-dependent DEG (Figure 1). The approaches used were, 1) Fold Change: the E-dependent DEG list was sorted according to fold change in expression, to identify the genes with the greatest up regulation and down regulation; 2) P-value: the E-dependent DEG list was sorted according to the statistical significance of the expression difference; 3) GeneGo analysis: 5 major GeneGo biological processes genes: Hormonal processes, Reproductive processes, Sex determination and differentiation, Apoptosis and, Cellular processes and 4) Genes containing EREs: Dragon ERE Locator version 6 was used to identify genes that contain ERE promoters from the shortlisted genes from step 1, 2 and 3. Using these criteria the list was reduced to generate a 78 E-dependent DEG with ERE Shortlist (Table S2). The results of each sorting method (1–4) used in the generation of the E-dependent DEG with ERE Shortlist are detailed below:

#### 1. Genes from the E-dependent DEG list showing greatest fold change in expression

The twenty genes found to be most up regulated and the twenty found to be most down regulated in the DEG List based upon fold change values are summarised in Table 2 and Table 3, respectively. The fold change values range from 47 to 6.3 for the most up regulated genes and from −15.7 to −4.8 for the most down regulated genes.

**Table 2:** Twenty most up-regulated genes in the E-dependent DEG list.

	Genbank	Gene symbol	Name	Fold change	P-value
1	NM_178396	Car12*	carbonic anyhydrase 12	**47.04**	0.00076
2	NM_008491	Lcn2*	lipocalin 2	**20.61**	0.00358
3	XM_130383	8030411F24Rik*	RIKEN cDNA 8030411F24	**13.46**	0.00144
4	XM_485085	Defb36*	defensin beta 36	**13.39**	0.00125
5	NM_011413	C4* ^†^	sex-limited protein	**12.93**	0.00198
6	XM_355911	Pace4	paired basic amino acid cleaving system 4 isoform 1	**11.18**	0.00008
7	NM_008770	Cldn11*	claudin 11	**10.89**	0.00001
8	XM_357518	LOC384244*	similar to Forkhead box protein L1 (Forkhead-related protein FKHL11) (Forkhead-related transcription factor 7) (FREAC-7)	**10.87**	0.00255
9	XM_130716	Gm122*	hypothetical protein LOC209351 isoform 1	**9.23**	0.00438
10	NM_009778	C3	complement component 3	**8.91**	0.00205
11	NM_009979	Cst9*	cystatin 9	**8.73**	0.00007
12	NM_024285	Bves*	blood vessel epicardial substance	**8.10**	0.00720
13	NM_009252	Serpina3n*	serine (or cysteine) proteinase inhibitor, clade A, member 3N	**7.90**	0.01640
14	NM_144936	BC018222*	transmembrane protein 45b	**7.89**	0.00092
15	NM_026323	Wfdc2	WAP four-disulfide core domain 2	**6.84**	0.00251
16	NM_009301	Svs5*	seminal vesicle secretion 5	**6.67**	0.00285
17	NM_029325	Spinlw1*	eppin	**6.65**	0.00051
18	NM_007878	Drd4	dopamine receptor 4	**6.34**	0.00205
19	XM_133614	Kctd14	potassium channel tetramerisation domain containing 14	**6.27**	0.0141
20	NM_007739	Col8a1*	procollagen, type VIII, alpha 1	**6.27**	0.00854

Cut-off is 2 fold change in expression in ArKO compared to WT (n = 3), p-value≤0.05.

*denote genes have not been shown to express in the ovary.

**Table 3:** Twenty most down-regulated genes in the E-dependent DEG list.

	Genbank	Gene symbol	Name	Fold change	P-value
1	NM_134066	Akr1c18*	aldo-keto reductase family 1, member C18	**−15.67**	0.00634
2	NM_144890	BC018465*	hypothetical protein LOC228802	**−11.96**	0.00195
3	NM_007409	Adh1*	alcohol dehydrogenase 1 (class I)	**−11.66**	0.00031
4	NM_010476	Hsd17b7	hydroxysteroid (17-beta) dehydrogenase 7	**−10.83**	0.01950
5	NM_176930	C130076O07Rik*	neuronal cell adhesion molecule	**−10.16**	0.00779
6	NM_013582	Lhcgr	luteinizing hormone/choriogonadotropin receptor	**−9.62**	0.00012
7	NM_009285	Stc1	stanniocalcin 1	**−8.93**	0.00929
8	NM_175293	D630023F18Rik*	hypothetical protein LOC98303	**−6.99**	0.00062
9	NM_175556	A930027K05Rik*	phospholipase C-like 4	**−6.94**	0.01060
10	NM_199303	Bpil3*	bactericidal/permeability-increasing protein-like 3 precursor	**−6.71**	0.02000
11	NM_009610	Actg2	actin, gamma 2, smooth muscle, enteric	**−6.29**	0.00215
12	XM_129987	Ccdc3*	coiled-coil domain containing 3	**−6.25**	0.00030
13	NM_023635	Rab27a*	RAB27A protein	**−6.21**	0.04000
14	NM_144551	Trib2*	tribbles homolog 2	**−5.99**	0.00013
15	NM_130450	Elovl6*	ELOVL family member 6, elongation of long chain fatty acids	**−5.38**	0.00170
16	NM_019517	Bace2*	beta-site APP-cleaving enzyme 2	**−5.13**	0.04830
17	NM_022315	Smoc2*	secreted modular calcium-binding protein 2	**−5.10**	0.00359
18	NM_027865	Tmem25*	transmembrane protein 25	**−5.08**	0.00694
19	NM_001004365	Arp3b*	ARP3 actin-related protein 3 homolog B	**−5.08**	0.00020
20	NM_011797	Car14*	carbonic anhydrase 14	**−4.83**	0.00004

Cut-off is 2 fold change in expression in ArKO compared to WT (n = 3), p-value≤0.05.

*denote genes have not been shown to express in the ovary.

#### 2. Genes from the E-dependent DEG list showing the most significant changes in gene expression

The twenty genes found to be most significantly up regulated compared to control and the twenty found to be most significantly down regulated compared to control, based upon the lowest p-values, are summarised in Table 4 and Table 5, respectively. The p-values range from 0.000001 to 0.000214 for the most up regulated genes and from 0.000004 to 0.000203 for the most down regulated genes.

**Table 4:** Twenty most significant differentially up-regulated genes in the E-dependent DEG list.

	Genbank	Gene symbol	Name	Fold change	P-value
1	NM_145219	Lgi3*	leucine-rich repeat LGI family, member 3	5.70	**0.000001**
2	NM_008770	Cldn11*	claudin 11	10.89	**0.000006**
3	NM_009893	Chrd	chordin	2.28	**0.000016**
4	AK033210	8030463A06Rik*	hypothetical protein LOC414083	2.25	**0.000020**
5	XM_148974	BC034090*	hypothetical protein XP_148974 isoform 1	2.11	**0.000024**
6	NM_021564	Fetub*	fetuin beta	2.91	**0.000032**
7	NM_177547	Cisk; 2510015P22Rik; A330005P07Rik*	serum/glucocorticoid regulated kinase 3	2.02	**0.000046**
8	NM_009979	Cst9*	cystatin 9	8.73	**0.000067**
9	NM_028295	Pdir*	protein disulfide isomerase associated 5	5.43	**0.000075**
10	XM_355911	Pace4	paired basic amino acid cleaving system 4 isoform 1	11.18	**0.000078**
11	XM_129211	Psat1*	phosphoserine aminotransferase 1	2.49	**0.000081**
12	NM_018784	Siat10*	alpha2,3-sialyltransferase VI	2.13	**0.000087**
13	NM_031380	Fstl3	follistatin-like 3	3.00	**0.000090**
14	XM_110968	D11Ertd686e*	dynein, axonemal, heavy chain 9 isoform 1	3.13	**0.000110**
15	NM_013589	Ltbp2	latent transforming growth factor beta binding protein 2	2.93	**0.000118**
16	NM_029173	4930519N16Rik*	nucleoredoxin	2.54	**0.000138**
17	NM_008968	Ptgis	prostaglandin I2 (prostacyclin) synthase	3.75	**0.000153**
18	NM_177547	Cisk; 2510015P22Rik; A330005P07Rik*	serum/glucocorticoid regulated kinase 3	2.08	**0.000163**
19	NM_007752	Cp*	ceruloplasmin	4.66	**0.000214**
20	NM_011594	Timp2	tissue inhibitor of metalloproteinase 2	3.00	**0.000214**

Genes up regulated in the E-dependent DEG list were ranked from most significant to least significant according to fold change. Cut-off is 2 fold change in expression in ArKO compared to WT (n = 3), p-value ≤0.05.

*denote genes have not been shown to express in the ovary.

**Table 5:** Twenty most significant differentially down-regulated genes in the E-dependent DEG list.

	Genbank	Gene symbol	Name	Fold change	P-value
1	NM_176950	9230107O10Rik*	hypothetical protein LOC319579	−2.58	**0.000004**
2	NM_010417	Heph*	hephaestin isoform 1	−2.54	**0.000020**
3	NM_019919	Ltbp1*	latent transforming growth factor beta binding protein 1 isoform a	−3.23	**0.000036**
4	NM_011797	Car14*	carbonic anhydrase 14	−4.83	**0.000036**
5	NM_010544	Ihh	Indian hedgehog	−2.98	**0.000113**
6	NM_013582	Lhcgr	luteinizing hormone/choriogonadotropin receptor	−9.62	**0.000117**
7	NM_153546	Oact1*	O-acyltransferase (membrane bound) domain containing 1	−3.17	**0.000118**
8	AK080168	ENSMUSG00000055015*		−3.27	**0.000118**
9	XM_358333	E030049G20Rik*	Nck-associated protein 5	−2.16	**0.000119**
10	NM_011146	Pparg	peroxisome proliferator activated receptor gamma	−2.83	**0.000119**
11	NM_144551	Trib2*	tribbles homolog 2	−5.99	**0.000131**
12	NM_007904	Ednrb	endothelin receptor type B	−3.33	**0.000148**
13	NM_025363	1110001J03Rik*	hypothetical protein LOC66117	−2.61	**0.000151**
14	NM_011535	Tbx3*	T-box 3 protein isoform 1	−3.42	**0.000163**
15	NM_010497	Idh1	isocitrate dehydrogenase 1 (NADP+), soluble	−2.74	**0.000164**
16	NM_181990	Cklfsf2a*	chemokine-like factor super family 1	−2.78	**0.000175**
17	NM_019739	Foxo1	forkhead box O1a	−2.87	**0.000176**
18	NM_008289	Hsd11b2	hydroxysteroid 11-beta dehydrogenase 2	−2.11	**0.000177**
19	NM_001004365	Arp3b*	ARP3 actin-related protein 3 homolog B	−5.08	**0.000202**
20	NM_010570	Irs1	insulin receptor substrate 1	−2.16	**0.000203**

Genes down regulated in the E-dependent DEG list were ranked from most significant to least significant according to fold change. Cut-off is 2 fold change in expression in ArKO compared to WT (n = 3), p-value ≤0.05.

*denote genes have not been shown to express in the ovary.

#### 3. GeneGo

Having narrowed the E target genes identified using the Illumina microarray down to a workable shortlist of 78 E-dependent DEG with ERE (Table S2); the molecular interactions between shortlisted genes were determined. Of the total set of 78 genes, ∼37 (∼47%) were not previously documented as ovarian genes, as determined by an extensive literature review and cross-checking with online databases (PubMed and Ovarian Kaleidoscope Database). Therefore, the identification of known and unknown genes could reveal how E affects regulation in folliculogenesis. We used GeneGo Pathways Analysis software to analyse the E-dependent DEG Shortlist and create ‘networks’ best connecting the 78 unique genes.

**Figure 3:**
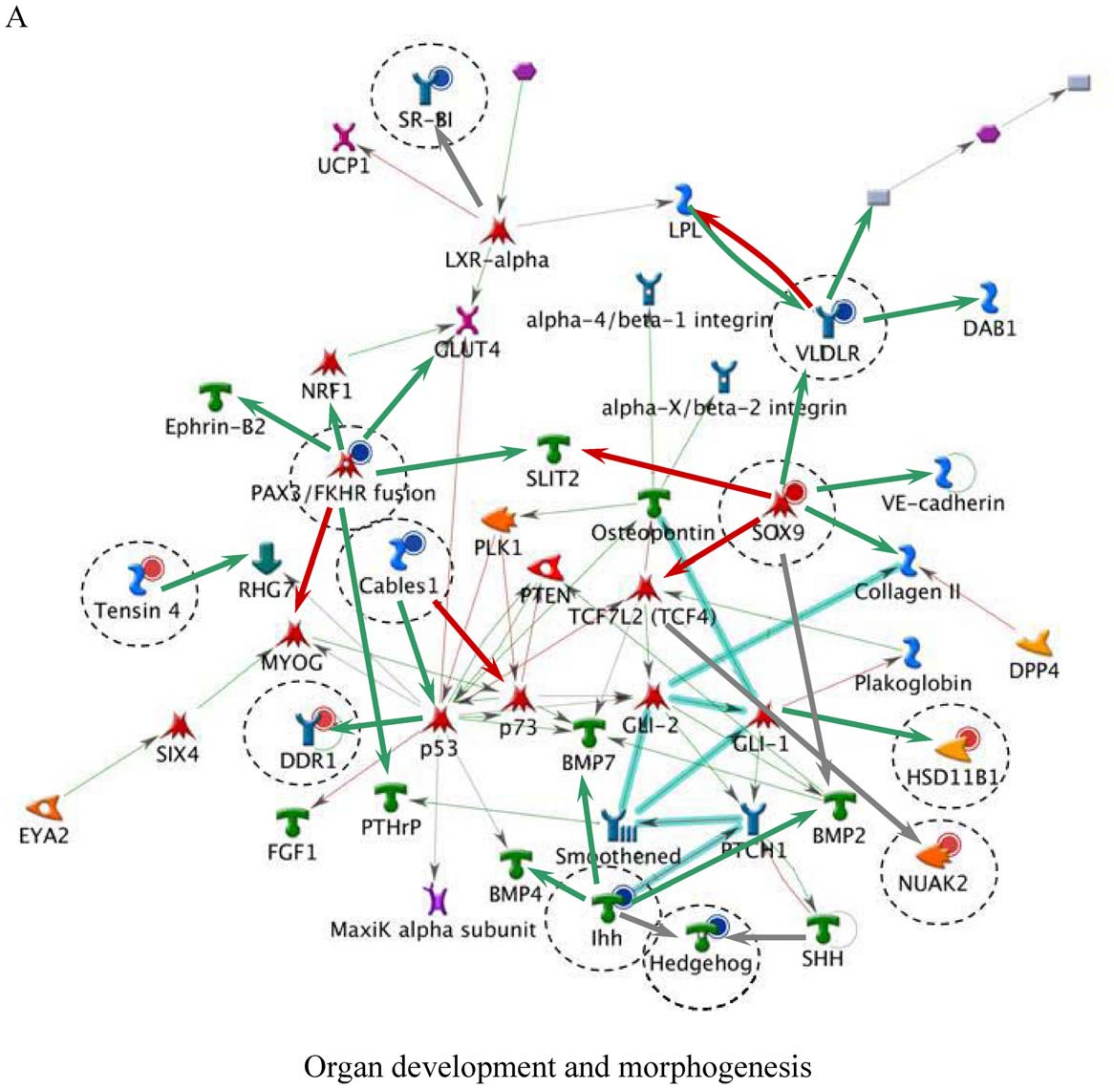
Molecular network of identifying E-dependent DEG with ERE Shortlist genes in ArKO ovary. Solid connecting lines represent a direct relationship between two molecules: activation marked as green solid line, inhibition marked as red solid line and unspecified marked as gray solid line. Thick cyan lines indicate the fragments of canonical pathways. Up-regulated genes are marked with red circles; down-regulated with blue circles. Genes with ERE are encircled. (Please refer to Figure S1 for detailed network object legend).

**Figure 4:**
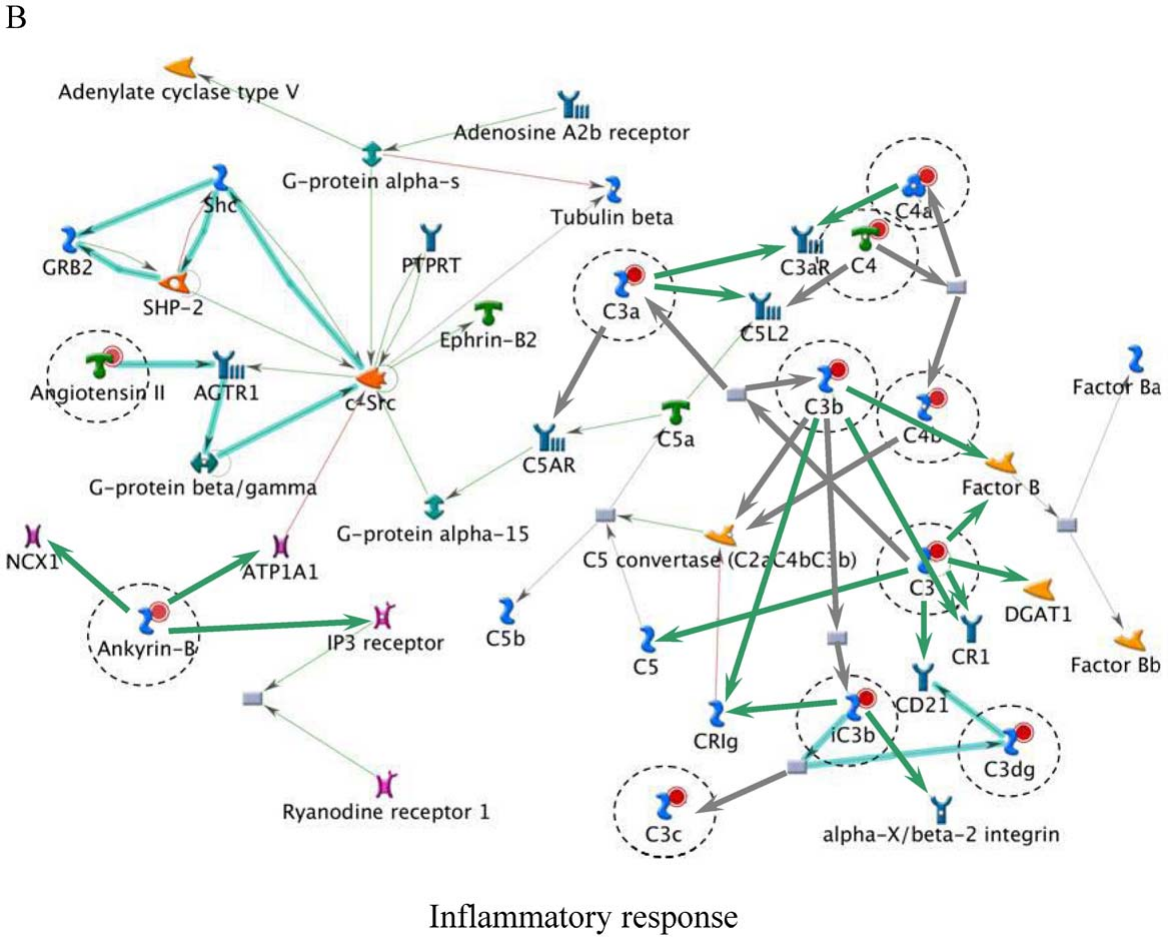
Molecular network of identifying E-dependent DEG with ERE Shortlist genes in ArKO ovary. Solid connecting lines represent a direct relationship between two molecules: activation marked as green solid line, inhibition marked as red solid line and unspecified marked as gray solid line. Thick cyan lines indicate the fragments of canonical pathways. Up-regulated genes are marked with red circles; down-regulated with blue circles. Genes with ERE are encircled. (Please refer to Figure S1 for detailed network object legend).

**Figure 5:**
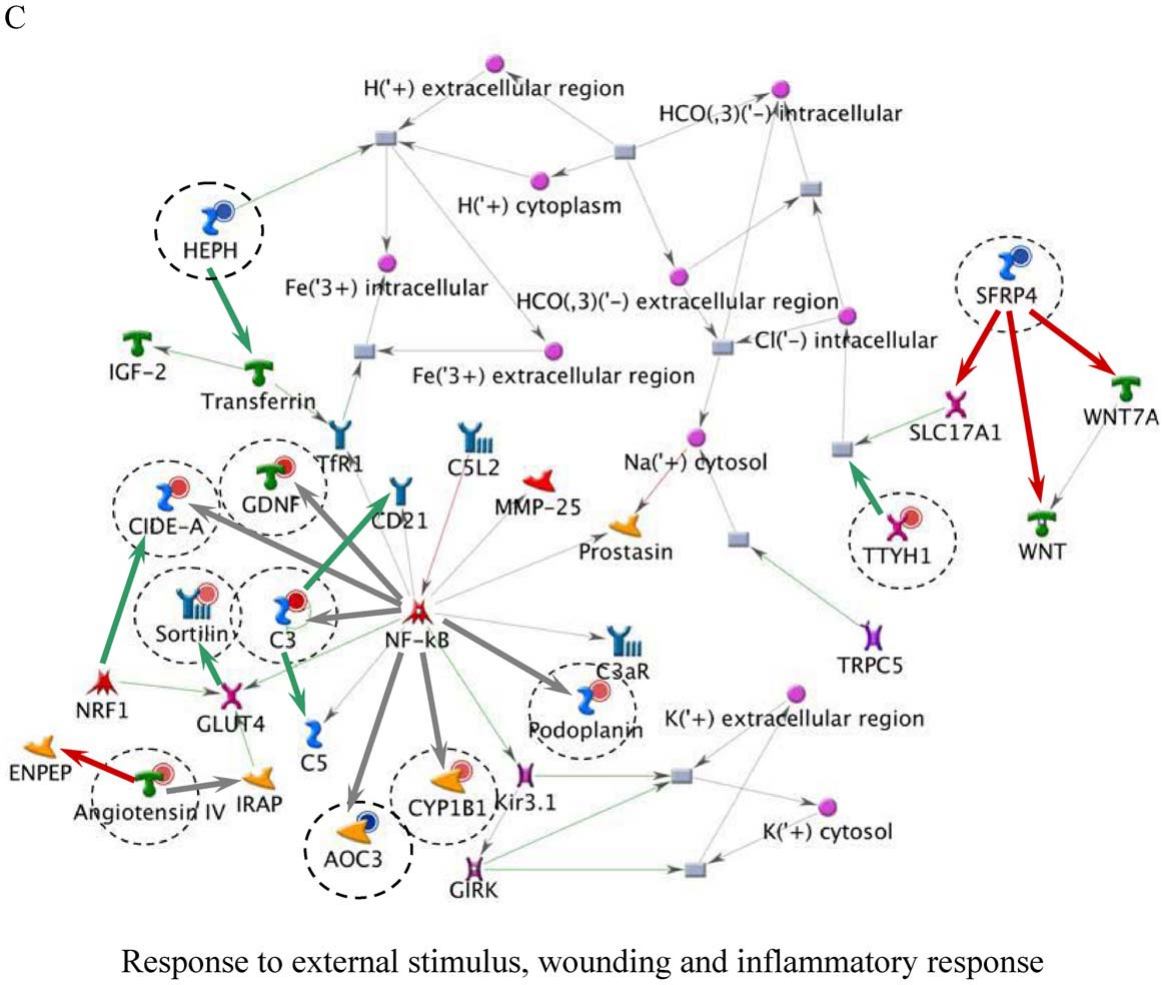
Molecular network of identifying E-dependent DEG with ERE Shortlist genes in ArKO ovary. Solid connecting lines represent a direct relationship between two molecules: activation marked as green solid line, inhibition marked as red solid line and unspecified marked as gray solid line. Thick cyan lines indicate the fragments of canonical pathways. Up-regulated genes are marked with red circles; down-regulated with blue circles. Genes with ERE are encircled. (Please refer to Figure S1 for detailed network object legend).

**Figure 6:**
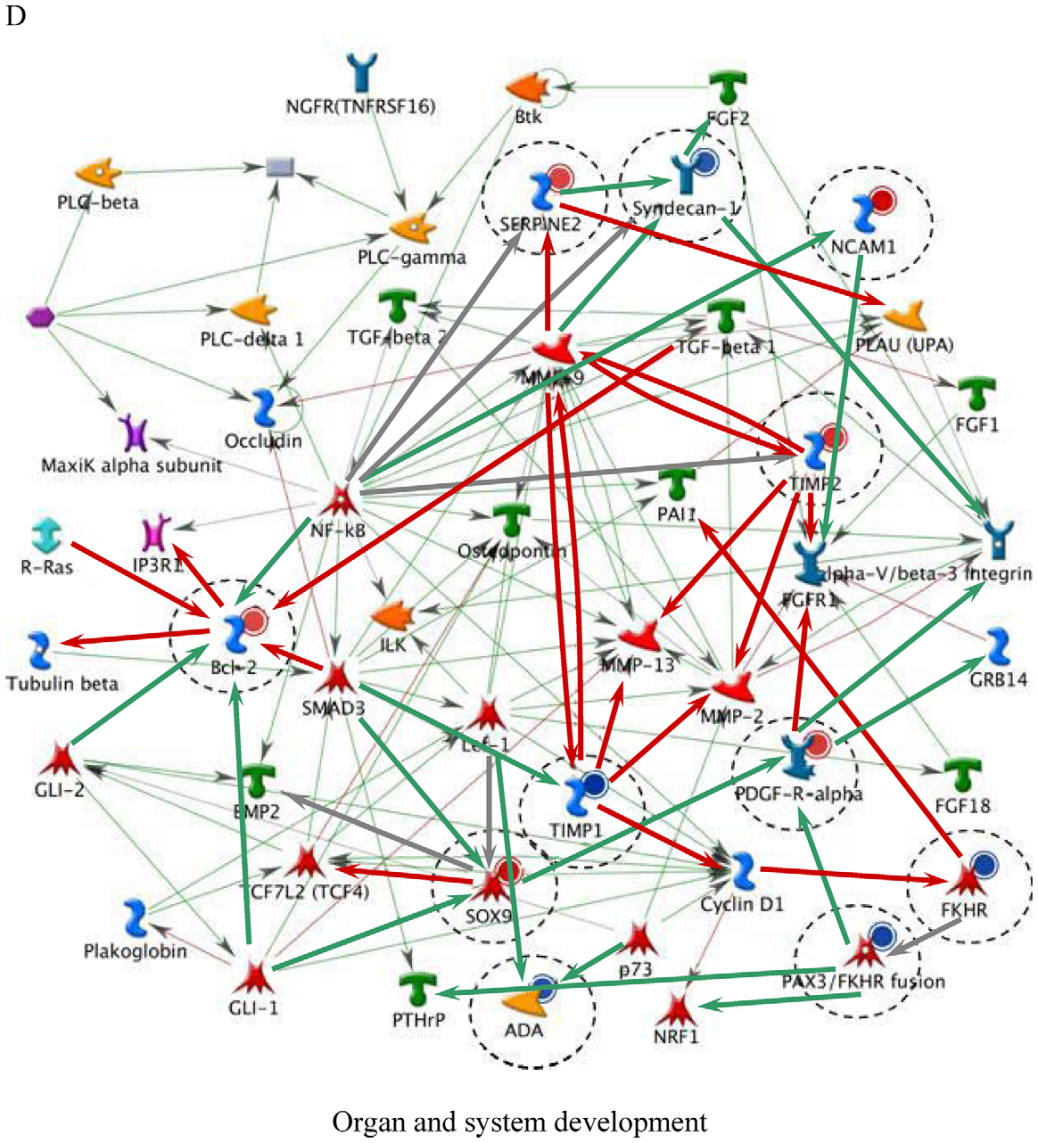
Molecular network of identifying E-dependent DEG with ERE Shortlist genes in ArKO ovary. Solid connecting lines represent a direct relationship between two molecules: activation marked as green solid line, inhibition marked as red solid line and unspecified marked as gray solid line. Thick cyan lines indicate the fragments of canonical pathways. Up-regulated genes are marked with red circles; down-regulated with blue circles. Genes with ERE are encircled. (Please refer to Figure S1 for detailed network object legend).

**Figure 7:**
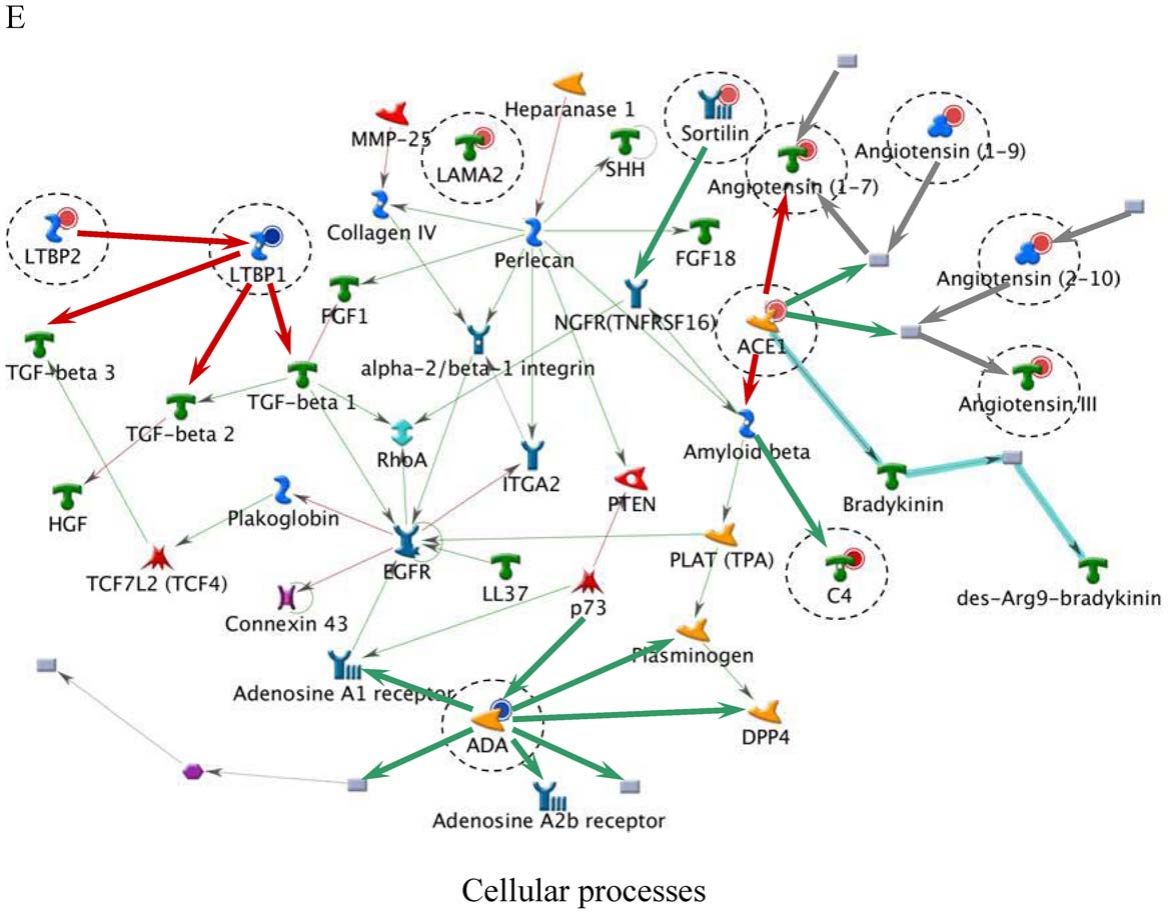
Molecular network of identifying E-dependent DEG with ERE Shortlist genes in ArKO ovary. Solid connecting lines represent a direct relationship between two molecules: activation marked as green solid line, inhibition marked as red solid line and unspecified marked as gray solid line. Thick cyan lines indicate the fragments of canonical pathways. Up-regulated genes are marked with red circles; down-regulated with blue circles. Genes with ERE are encircled. (Please refer to Figure S1 for detailed network object legend).

Table 6 lists the top five most relevant networks identified by the GeneGo software. All the molecules listed within each network are connected based upon their known relationships or functions within the GeneGo Pathways Knowledge Base. The gene content of the E-dependent DEG Shortlist is used as the input list for generation of biological networks using the Analyze Networks (AN) algorithm. This is a variant of the shortest paths algorithm with main parameters of 1) relative enrichment with the E-dependent DEG Shortlist, and 2) relative saturation of networks with canonical pathways (g-Score). These networks are built upon the E-dependent DEG Shortlist and unique for the E-dependent DEG Shortlist (Detailed network object legend, Figure S1). For instance, Network A (Figure 3) with the highest g-Score (43.64) (Table 6) contained 11 genes. The top processes associated with this network included ‘organ development’, ‘anatomical structure morphogenesis’ and ‘organ morphogenesis’. Both Network B and C (Figure 4 and 5) were noted as having genes that are related to ‘response to stimulus’ and ‘inflammatory responses’. Similarly, Network D (Figure 6) further emphasised processes associated with organ development, system development and anatomical structure development. Network E (Figure 7) was associated with cellular processes, such as, regulation of multicellular organismal process, regulation of cell migration and regulation of cellular component movement. All Networks: A to E (Figure 3, 4, 5, 6, 7) complemented processes that were identified in the GO analysis.

**Table 6:** Top five gene networks identified by GeneGo Pathways Analysis using the E-dependent DEG Shortlist of 78 unique genes.

	Processes	p-value	z-Score	g-Score
A	Organ morphogenesis (58.7%), organ development (73.9%), anatomical structure morphogenesis (63.0%)	1.56e-21	39.89	43.64
B	Complement activation (23.3%), activation of plasma proteins involved in acute inflammatory response (23.3%), response to stimulus (86.7%)	1.59e-19	37.44	41.19
C	Response to external stimulus (50.0%), response to wounding (40.0%), inflammatory response (33.3%)	1.20e-21	40.32	40.32
D	Organ development (79.2%), system development (85.4%), anatomical structure development (85.4%)	2.01e-21	39.47	39.47
E	Regulation of multicellular organismal process (64.7%), regulation of cell migration (38.2%), regulation of cellular component movement (38.2%)	2.51e-17	34.06	35.31

The molecules that make up the top 5 Networks (A–E) identified using the E-dependent DEG with ERE Shortlist and ranked by GeneGo according to the g-Score are shown. The g-Score modifies the z-Score based on the number of Canonical Pathways used to build the network. If a network has a high g-Score, it is saturated with expressed genes (from z-Score) and it contains many Canonical Pathways. Sorting the table by this value essentially enables you to sort the table by two factors at once. The number of genes involved within the processes is shown in percentage.

## Discussion

In this study, a genome wide transcriptional profiling microarray analysis was used for the first time to specify E-dependent genes or associated targets of E action in the mouse ovary. First, our studies led us to identify a set of 450 E-dependent ovarian genes whose biological roles may have significant relevance to functional and structural development of the ovary in mice. Second, we identified a set of 78 of these genes were most likely to have direct biological E targets and out of these, approximately 37 genes were not previously described in the ovary. Female ArKO mice are infertile due to failure to ovulate [14]. They have elevated levels of circulating gonadotrophins and testosterone, severely underdeveloped uteri [14], [15], [16] and increased adiposity [17], [18]. The ovaries possess hemorrhagic cysts and apparent sex-reversal of the ovarian somatic cells; that is granulosa cells become Sertoli-like cells [8], [20], [21]. The ArKO ovaries also displayed an infiltration of macrophages and contain substantial collagen deposition, a characteristic of tissues that are fibrotic [14]. It was logical to ask the question: were there changes in genes associated with these processes?

### Masculinization

One of the major genes associated with masculinization, Sox9 [19] was confirmed to be expressed in the ArKO ovary from our microarray analysis results. Early in gonad development soon after the expression of SRY begins, *Sox9* expression is strongly upregulated in Sertoli cells; Sox9 expression is maintained in the testis, whereas it is downregulated in the ovary [21]. The presence of the Sertoli cell marker *Sox9* in the ArKO ovary confirmed the presence of testicular cell-like cells [19], [20]. 17α-Hydroxylase (*17α-OHase*) and 17β-Hydroxysteroid Dehydrogenase type-3 (*17β-Hsd3*, the Leydig cell- specific) are two important steroidogenic enzymes in the production of testosterone. ArKO mice also showed increased levels of *17α-OHase* and *17β-Hsd3* at 10 weeks of age, when Sertoli- and Leydig-like cells are beginning to populate the ovary [19] which were correlated with high serum testosterone levels [8]. Based on the known mechanisms maintaining Sertoli cell development and function in the normal (Wt testis), we identified two very intriguing novel targets of E that were significantly increased in the ArKO ovary, Claudin-11 (*Cldn11*) and platelet-derived growth factor receptor α (*Pdgfrα*). *Cldn11* is a protein component in tight junctions between Sertoli cells which are important for the maintenance of the blood–testis barrier [22,23] and *Pdgfrα* was identified previously as a key player downstream of *Sry* in testis organogenesis and Leydig cell differentiation [24]. In the ArKO ovary, granulosa cells become Sertoli-like cells [8], [19], [20] and interstitial cells were identified ultrastructurally as Leydig cells [19]. However, to date, the exact role of E in the somatic cell trans/de-differentiation is unclear, although the fact that granulosa and interstitial cells express ER [25] points to a direct action on the gonadal somatic cells to maintain a ovarian phenotype. It is possible that the oocyte, which also expresses ER [26], plays a part in maintaining the ovarian phenotype with many recorded cases of premature oocyte loss leading to transdifferentiation of granulosa cells into Sertoli-like cells [8], [19]. We therefore, hypothesized that E actively down regulates genes involved in masculinization to suppress the testicular phenotype, and that this action could be either direct on the somatic cells or via the oocyte or both.

### Folliculogenesis

From the morphological and stereological assessments made in previous studies [14], [19], follicular growth beyond the preantral stage is unsuccessful in the absence of E, with most succumbing to atresia, developing into hemorrhagic cysts or taking on a testicular phenotype (as discussed above). We hypothesized that this phenomenon occurs due to the loss of oocytes in the ArKO ovary and this was supported by the array of genes that were identified in this study. For example, Paired basic amino acid cleaving system 4 isoform 1 (*Pace4*) or Proprotein convertase subtilisin/kexin type 6 (*Pcsk6*) is a proprotein convertase required for producing active forms of important TGFβ ligands in the ovary [27]. The levels of this enzyme were increased in the ArKO ovary. Steady state mRNA levels of *Pcsk6* in granulosa cells are normally required to be suppressed by the oocyte during preantral to antral follicle transition [28]. Thus, Pcsk6 represents another granulosa cell transcript regulated by oocytes as well as E, but exhibiting a novel expression pattern during follicular development. Cytochrome P450 lanosterol 14 alpha-demethylase (*Cyp51*), which was decreased in the ArKO ovary, has long been known for reactions involved in drug metabolism and synthesis of cholesterol, steroids and other lipids. Recent studies on *Cyp51* show that it promotes the initiation of meiosis in early folliculogenesis [29], [30]. The decrease in expression of Cyp51 in the ArKO ovary implies that E has a role in controlling early follicular development. This observation is also supported in primate studies by Zachos *et al.* [31], in which fetal baboons deprived of E showed a 50% reduction in the number of primordial follicles which was restored by E_2_ administration. Similar data for the ArKO mouse ovary has not been reported, although it has been shown that E influences the breakdown of oocyte nests prior to the formation of primordial follicles [32], [33].

### Novel Network Processes

A significant over representation of genes involved in organ development and morphogenesis, inflammatory responses and cellular processes were identified in both the Gene Ontology annotation and GeneGo Pathway analyses of differentially expressed genes in ArKO ovary.

#### 1. Hedgehog signalling

One of the affected pathways in the ArkO ovary is the Hedgehog-Patched (HH) signalling pathway, which is involved in ovarian follicle development. Expression of HH ligands, Sonic hedgehog (*Shh*), Indian hedgehog (*Ihh*) and Desert hedgehog (*Dhh*) are developmentally regulated in the adult ovary [36]. Other components of the HH pathway such as Hedgehog receptors (*PTCH1* and *PTCH2*) and signal transducer Smoothened (*SMO*) are expressed by granulosa cells of the follicle [34]. Hedghog signalling in the mammalian ovary plays a role in the communication between granulosa cells and the developing theca cells [35]. A recent study by Ren and colleagues [36], showed that Amhr2^cre/+^SmoM2 mutant mice that constitutively expresses the dominant active form of *SMO* in the ovary, were anovulatory because of a dramatic reduction in the muscle cells of the theca layer surrounding the developing follicles. These muscle cells within the theca layer play a role in the release of the oocyte at the time of ovulation. One of the smooth muscle markers, actin, gamma 2, smooth muscle, enteric (*Actg2*) was found to be significantly decreased in the Amhr2^cre/+^SmoM2 mutant mice [36] and this was also the case in the ArKO ovary (Table 4), suggesting E might play a role in regulating the smooth muscle cells within the ovary during ovulation. In our study, *Ihh* mRNA expression was found to be decreased in the ArKO mouse ovary (Table 4). The reduced *Ihh* production in the ArKO ovary implies an inhibition of HH signalling and a hormonal regulation of the Hedgehog system (Figure 3).

#### 2. Inflammatory response and fibrosis

It was not surprising to see the inflammatory responses network filling two of the top 5 network processes (Figure 4 and 5) in the ArKO ovary. Previous studies have shown an increased infiltration of macrophages and deposition of collagen in the ArKO ovary, suggesting that in the absence of E, the ArKO ovary begins to degenerate [19]. Mast cells, macrophages and granulocytes usually accumulate in rat ovaries during the preovulatory period [37]. Their presence in ArKO ovaries indicates that the chemokine signals for ovulation including an increase in the abundance of the immune mediators have been activated, but the follicles have been unable to respond. The increased deposition of collagen in the absence of E suggests that matrix metalloproteinases (MMPs), including the collagenases, have not been activated. This was reflected in the network processes (Figure 6), as *MMP-2*, *MMP-9*, tissue inhibitors of MMP 1 (*TIMP1*) and *TIMP2* were either directly or indirectly affected in the absence of E. Detailed studies on the hormonal regulation, site of synthesis, and absolute requirement on individual MMPs and TIMPs, for successful ovulation remain to be elucidated. The improved ovarian morphology in ArKO mice treated with E [8], correlated with a decrease in macrophages, mast cells and collagen again implicating the direct or indirect effects of E on the extracellular matrices of the ovary.

#### 3. WNT signalling

WNT signaling influences cellular differentiation and proliferation in the embryonic and adult ovaries. In this study, we identified a significant decrease in Secreted frizzled-related protein 4 (*SFRP4*) mRNA expression in the ArKO mouse ovary. *SFRP4* is a member of the Frizzled like cystein rich domain family and a modulator of non canonical WNT signalling [38]. It has been shown that *SFRP4* transcripts co-localize in the ovary to sites of *Fz-1*, *Wnt4*, and *Fz-4* expression, suggesting that *SFRP4* may regulate signaling through these factors in the ovary [38], [39], [40]. Both *SFRP4* and *Fz-1* are expressed in granulosa cells of large follicles suggesting a potential regulation by SFRP-4 of *Fz-1* signals that may impact granulosa cell differentiation or expression of genes involved in follicle rupture and ovulation [38], [40]. *Wnt4* is a member of the WNT family of intracellular growth and differentiation factors, which regulate several key developmental steps. Deficiency of *Wnt4* leads to partial female to male sex reversal and a marked reduction in oocyte number [41]. In addition, *Fz-4* deficient mice showed defective corpora lutea [42]. It is therefore, tempting to speculate that E is regulating *SFRP4* to maintain the ovarian phenotype, The ArKO model will be a useful tool to further study the effect of loss of estrogen on WNT signaling.

### Other genes of interest

This study identified genes with known or suspected roles in ovarian functions as stated above. Interestingly, there were 37 genes from the E-dependent DEG Shortlist that possessed EREs that were completely novel to ovarian function. The roles of these genes in ovarian function will require further research and verification. There were also genes (*DAX1* [8], *MIS* [8], *ERα*, [8], *ERβ* [8], *GDF9* [43], and *Cyclin D2* [5]) that were expected to be regulated (up or down) in the absence of E as a result of previous studies, which were not detected in the present study. It is likely that some genes will be missed, because they may be affected by E at earlier time-points or their expression may be below the level of detection in this study.

### Conclusion

Our success in identifying E-dependent ovarian genes validates the usefulness of whole-genome arrays. We identified more than double the number of E-dependent ovarian/oocyte factors previously reported. The E-dependent ovarian genes identified in this study provide attractive candidates for studying female infertility [44]. In addition, analyses can be easily expanded by inclusion of additional expression profiles, such as, a set of E_2_ replacement ArKO ovarian genes or refined by identifying which ER subtype is responsible for specific E-dependent ovarian genes. Such study will be required in order to define more precisely mechanisms of ovarian function, as well as provides new targets for the development of contraceptives and highlights genes previously uncharacterised in the ovary, that may be involved in the pathophysiology of female reproductive disorders. Furthermore, these E-dependent expression datasets will enable further gene discovery focusing on microRNAs and non-coding RNAs.

## Supporting Information

Table S1 E-dependent DEG list.
(0.91 MB DOC)

Table S2 78 most significant differentially genes that are most likely to represent direct biological E target genes.
(0.13 MB DOC)

Figure S1 Network object legend.
(3.18 MB TIF)
