# Supplementary material for: Estrogen-Dependent Gene Expression in the Mouse Ovary
Source: PLoS One. 2011 Feb 9;6(2):e14672. doi: 10.1371/journal.pone.0014672 (PMC3036593; doi:10.1371/journal.pone.0014672)
Supplement: Table S1 — E-dependent DEG list. (0.91 MB DOC) [file pone.0014672.s001.doc]

Supplemental Table 2. E-dependent DEG list

|  | **Illumina Gene Name** | **Product** | **Common** | **Gene Symbol** | **Genbank** |
| --- | --- | --- | --- | --- | --- |
| 1 | scl36787.11_464-S | carbonic anyhydrase 12 | Car12 | Car12 | NM_178396 |
| 2 | scl19446.4_4-S | lipocalin 2 | Lcn2 | Lcn2 | NM_008491 |
| 3 | scl20156.3.1_46-S | RIKEN cDNA 8030411F24 | 8030411F24Rik |  | XM_130383 |
| 4 | scl0266620.1_2-S | defensin beta 36 | Defb36 |  | XM_485085 |
| 5 | scl020567.3_14-S | sex-limited protein | C4 | C4a | NM_011413 |
| 6 | scl32572.22_334-S | paired basic amino acid cleaving system 4 isoform 1 | Pace4 | Pcsk6 | XM_355911 |
| 7 | scl23301.3_380-S | claudin 11 | Cldn11 | Cldn11 | NM_008770 |
| 8 | GI_38081367-S | similar to Forkhead box protein L1 (Forkhead-related protein FKHL11) (Forkhead-related transcription factor 7) (FREAC-7) | LOC384244 | EG384244 | XM_357518 |
| 9 | GI_20861622-S | hypothetical protein LOC209351 isoform 1 | Gm122 |  | XM_130716 |
| 10 | scl49743.39.1_15-S | complement component 3 | C3 | C3 | NM_009778 |
| 11 | scl20152.3.1_18-S | cystatin 9 | Cst9 | Cst9 | NM_009979 |
| 12 | scl38950.5_617-S | blood vessel epicardial substance | Bves | Bves | NM_024285 |
| 13 | scl020716.5_261-S | serine (or cysteine) proteinase inhibitor, clade A, member 3N | Serpina3n | Serpina3n | NM_009252 |
| 14 | scl36063.7.1_2-S | transmembrane protein 45b | BC018222 | Tmem45b | NM_144936 |
| 15 | scl19931.7.1_28-S | WAP four-disulfide core domain 2 | Wfdc2 | Wfdc2 | NM_026323 |
| 16 | scl19941.3.19_3-S | seminal vesicle secretion 5 | Svs5 | Svs5 | NM_009301 |
| 17 | scl18357.4.1_225-S | eppin | Spinlw1 | Spinlw1 | NM_029325 |
| 18 | scl0013491.2_2-S | dopamine receptor 4 | Drd4 | Drd4 | NM_007878 |
| 19 | GI_38088807-S | potassium channel tetramerisation domain containing 14 | Kctd14 |  | XM_133614 |
| 20 | scl0012837.1_129-S | procollagen, type VIII, alpha 1 | Col8a1 | Col8a1 | NM_007739 |
| 21 | scl30661.9.1_0-S | zymogen granule membrane protein 16 | 1810010M01Rik | 1810010M01Rik | NM_026918 |
| 22 | scl48108.3_632-S | glial cell line derived neurotrophic factor | Gdnf | Gdnf | NM_010275 |
| 23 | scl48785.4.1_310-S |  |  |  | XM_148432 |
| 24 | scl0239435.2_82-S | alanine and arginine rich domain containing protein | Aard | Aard | NM_175503 |
| 25 | scl46124.8.1_10-S | leucine-rich repeat LGI family, member 3 | Lgi3 | Lgi3 | NM_145219 |
| 26 | scl075443.2_91-S |  |  |  | XM_489359 |
| 27 | scl42108.6.1_31-S | serine (or cysteine) proteinase inhibitor, clade A, member 6 | Serpina6 | Serpina6 | NM_007618 |
| 28 | scl48517.26.1_0-S | protein disulfide isomerase associated 5 | Pdir | Pdia5 | NM_028295 |
| 29 | scl47080.2.4_26-S | lymphocyte antigen 6 complex, locus D | Ly6d | Ly6d | NM_010742 |
| 30 | scl017228.2_50-S | mast cell protease 5 | Mcpt5 | Cma1 | NM_010780 |
| 31 | scl0329173.1_142-S | a disintegrin and metallopeptidase domain 23 preproprotein | AW046396 |  | NM_177836 |
| 32 | scl0017233.1_195-S | mast cell protease-like | Cma2 |  | NM_008573 |
| 33 | scl29649.6.1_19-S | LIM and cysteine-rich domains 1 | Lmcd1 | Lmcd1 | NM_144799 |
| 34 | scl21666.4.43_5-S | glutathione S-transferase, mu 6 | Gstm6 | Gstm6 | NM_008184 |
| 35 | scl00170442.1_8-S | butyrobetaine (gamma), 2-oxoglutarate dioxygenase 1 (gamma-butyrobetaine hydroxylase) | Bbox1 | Bbox1 | NM_130452 |
| 36 | scl23358.23.1_77-S | ceruloplasmin | Cp | Cp | NM_007752 |
| 37 | GI_7110612-S | glutathione S-transferase, mu 6 | Gstm6 | Gstm6 | NM_008184 |
| 38 | scl35883.23_148-S | neural cell adhesion molecule 1 | Ncam1 | Ncam1 | NM_010875 |
| 39 | scl51827.6.1_1-S | cell death-inducing DNA fragmentation factor, alpha subunit-like effector A | Cidea | Cidea | NM_007702 |
| 40 | scl017227.3_18-S | mast cell protease 4 | Mcpt4 | Mcpt4 | NM_010779 |
| 41 | scl20130.6_7-S | hypothetical protein LOC69698 | 2310046K01Rik | 2310046K01Rik | NM_027172 |
| 42 | scl46162.12.1_3-S | hypothetical protein LOC71145 | 4933425F03Rik | Scara5 | NM_028903 |
| 43 | scl0170745.21_29-S | X-prolyl aminopeptidase (aminopeptidase P) 2, membrane-bound isoform 1 | Xpnpep2 | Xpnpep2 | NM_178074 |
| 44 | GI_38090072-S |  |  |  | XM_147082 |
| 45 | scl074170.1_145-S |  |  |  |  |
| 46 | scl54724.3.1_13-S |  |  |  |  |
| 47 | scl40760.4_18-S | SRY-box containing gene 9 | Sox9 | Sox9 | NM_011448 |
| 48 | GI_38075048-S | hypothetical protein LOC380863 | Gm905 |  | XM_354769 |
| 49 | scl00109676.2_66-S | ankyrin 2, brain isoform 3 | Ank2 | Ank2 | NM_178655 |
| 50 | scl30018.2.1_40-S |  |  |  |  |
| 51 | scl0057776.1_221-S | tweety 1 isoform 1 | Ttyh1 | Ttyh1 | NM_021324 |
| 52 | scl21071.1.1_82-S |  |  |  |  |
| 53 | scl016336.1_104-S | insulin-like 3 | Jak3 | Insl3 | NM_013564 |
| 54 | scl39222.8.1_55-S | carbonyl reductase 2 | Cbr2 | Cbr2 | NM_007621 |
| 55 | scl011425.3_30-S | apolipoprotein C-IV | Apoc4 | Apoc4 | NM_007385 |
| 56 | scl0394435.7_126-S | UDP glucuronosyltransferase 1 family, polypeptide A6B | Ugt1a6 |  | NM_201410 |
| 57 | scl31001.17.1_88-S | solute carrier organic anion transporter family, member 2b1 | Slco2b1 | Slco2b1 | NM_175316 |
| 58 | scl20155.3.1_29-S | cystatin-related epididymal spermatogenic protein 3 | 1700006F03Rik | Cst12 | XM_130487 |
| 59 | scl019223.1_1-S | prostaglandin I2 (prostacyclin) synthase | Ptgis | Ptgis | NM_008968 |
| 60 | scl0227929.1_2-S | pleckstrin homology, Sec7 and coiled-coil domains, binding protein | Pscdbp | Pscdbp | NM_139200 |
| 61 | scl53221.10_351-S | RIKEN cDNA 9230117N10 | 9230117N10Rik | Il33 | NM_133775 |
| 62 | scl00394432.2_137-S | UDP glucuronosyltransferase 1 family, polypeptide A7C | Ugt1a6 |  | NM_201642 |
| 63 | scl21072.26_540-S | laminin gamma 3 | Lamc3 | Lamc3 | NM_011836 |
| 64 | scl53840.18.1_253-S | synaptotagmin-like 4 | Sytl4 | Sytl4 | NM_013757 |
| 65 | scl00170460.2_242-S | StAR-related lipid transfer protein 5 | Stard5 | Stard5 | NM_023377 |
| 66 | scl21665.7.1_0-S | similar to Glutathione S-transferase Yb-3 (Chain 4) (GST Yb3) (GST class-mu 3) isoform 2 | Gstm7 | Gstm7 | XM_359308 |
| 67 | scl35681.2_249-S | F-box and leucine-rich repeat protein 22 | Fbxl22 | Fbxl22 | NM_175206 |
| 68 | scl31506.4.1_20-S | hepcidin antimicrobial peptide 2 | 1810073K19Rik | Hamp2 | NM_183257 |
| 69 | scl0070019.1_250-S |  |  |  |  |
| 70 | scl21737.4_335-S | olfactomedin-like 3 | Olfml3 | Olfml3 | NM_133859 |
| 71 | scl50565.20_286-S | mannosidase 2, alpha 1 | Man2a1 | Man2a1 | NM_008549 |
| 72 | scl54892.5_229-S | hypothetical protein LOC74437 isoform 1 | 4933402E13Rik |  | XM_135885 |
| 73 | scl0078558.2_130-S | toll-associated serine protease | Htra3 | Htra3 | NM_030127 |
| 74 | scl0075443.1_227-S |  |  |  | XM_489359 |
| 75 | scl34490.14.1_30-S | carboxylesterase 3 | Ces3 | Ces3 | NM_053200 |
| 76 | scl45351.6.1_2-S | surfactant associated protein C | Sftpc | Sftpc | NM_011359 |
| 77 | scl17154.1.413_251-S |  |  |  |  |
| 78 | scl0001840.1_53-S | protein disulfide isomerase associated 5 | Pdir | Pdia5 | NM_028295 |
| 79 | scl43314.2_288-S | hypothetical protein LOC104943 | 9030611O19Rik | 9030611O19Rik | NM_027828 |
| 80 | scl27024.7_349-S | fascin homolog 1, actin bundling protein (Strongylocentrotus) purpuratus) | Fscn1 | Fscn1 | NM_007984 |
| 81 | scl0002118.1_28-S | glutathione S-transferase, mu 6 | Gstm6 | Gstm6 | NM_008184 |
| 82 | GI_72534649-S |  |  |  |  |
| 83 | scl19066.9.1_77-S | serine (or cysteine) proteinase inhibitor, clade G, member 1 | Serping1 | Serping1 | NM_009776 |
| 84 | scl38227.11_29-S | uronyl-2-sulfotransferase | Ust | Ust | NM_177387 |
| 85 | scl20002.16.7_10-S | lipopolysaccharide-binding protein | Lbp | Lbp | NM_008489 |
| 86 | scl0003910.1_15-S | vanin 3 | Vnn3 | Vnn3 | NM_011979 |
| 87 | scl0170460.6_30-S | StAR-related lipid transfer protein 5 | Stard5 | Stard5 | NM_023377 |
| 88 | scl17789.7.1_75-S | cytochrome P450, family 27, subfamily a, polypeptide 1 | Cyp27a1 | Cyp27a1 | NM_024264 |
| 89 | ri|1110055O21|ZA00008M11|AK027978|943-S | angiopoietin-like 1 | Angptl1 | Angptl1 | AK027978 |
| 90 | scl20131.9.1_36-S | angiopoietin 4 | Angpt4 | Angpt4 | NM_009641 |
| 91 | scl35460.23_457-S | chr3 synaptotagmin | D9Ertd280e | D9Ertd280e | NM_177775 |
| 92 | scl40071.17.1_51-S | dynein, axonemal, heavy chain 9 isoform 1 | D11Ertd686e | Dnahc9 | XM_110968 |
| 93 | scl0003896.1_252-S | insulin-like growth factor 1 isoform 1 | Igf1 | Igf1 | NM_010512 |
| 94 | scl18498.2.1_0-S | defensin beta 19 | Defb19 | Defb19 | NM_145157 |
| 95 | scl022041.3_13-S | transferrin | Trf | Trf | NM_133977 |
| 96 | scl34179.5.1_14-S | angiotensinogen | Agt | Agt | NM_007428 |
| 97 | scl47093.2_645-S | G protein-coupled receptor 20 | Gpr20 | Gpr20 | NM_173365 |
| 98 | scl094284.5_3-S | UDP glucuronosyltransferase 1 family, polypeptide A6A | Ugt1a6 |  | NM_145079 |
| 99 | scl00223272.2_246-S | integrin, beta-like 1 | Itgbl1 | Itgbl1 | NM_145467 |
| 100 | scl25839.9.1_1-S | hypothetical protein LOC231832 | BC019731 | BC019731 | NM_144914 |
| 101 | scl19548.10.1_41-S | ficolin A | Fcna | Fcna | NM_007995 |
| 102 | scl000907.1_9-S | a disintegrin and metallopeptidase domain 23 preproprotein | Adam23 | Adam23 | NM_011780 |
| 103 | scl021858.1_100-S | tissue inhibitor of metalloproteinase 2 | Timp2 | Timp2 | NM_011594 |
| 104 | scl38709.6.1_4-S | follistatin-like 3 | Fstl3 | Fstl3 | NM_031380 |
| 105 | scl17463.8_535-S | putative ethanolamine kinase | Etnk2 | Etnk2 | NM_175443 |
| 106 | scl22085.6.1_94-S | retinoic acid receptor responder (tazarotene induced) 1 | Rarres1 | Rarres1 | XM_130987 |
| 107 | scl23564.6_587-S | glycoprotein 38 | Gp38 | Pdpn | NM_010329 |
| 108 | scl33257.7.1_7-S | WAP four-disulfide core domain 1 | Wfdc1 | Wfdc1 | NM_023395 |
| 109 | scl33211.11.6_30-S | dipeptidase 1 (renal) | Dpep1 | Dpep1 | NM_007876 |
| 110 | scl45919.12_334-S | integrin, beta-like 1 | Itgbl1 | Itgbl1 | NM_145467 |
| 111 | scl46955.19_187-S | unc-84 homolog B | B230369L08Rik | Unc84b | NM_194342 |
| 112 | ri|4933426K21|PX00020B07|AK016936|1368-S |  | 4933426K21Rik | 4933426K21Rik | AK016936 |
| 113 | scl41132.3.1_4-S | extracellular proteinase inhibitor | Expi | Expi | NM_007969 |
| 114 | scl0078926.2_61-S | GAS2-related protein isoform alpha | Gas2l1 | Gas2l1 | NM_144560 |
| 115 | scl36733.11.1_17-S | meiosis-specific nuclear structural protein 1 | Mns1 | Mns1 | NM_008613 |
| 116 | scl0016997.2_276-S | latent transforming growth factor beta binding protein 2 | Ltbp2 | Ltbp2 | NM_013589 |
| 117 | scl25560.4.1_13-S | glycoprotein hormones, alpha subunit | Cga | Cga | NM_009889 |
| 118 | GI_38077788-S | hypothetical protein LOC106068 isoform 1 | 9330175B01Rik |  | XM_128267 |
| 119 | scl49314.9.1_24-S | fetuin beta | Fetub | Fetub | NM_021564 |
| 120 | scl0003097.1_18-S | eppin | Spinlw1 | Spinlw1 | NM_029325 |
| 121 | scl41156.3.1_26-S | chemokine (C-C motif) ligand 8 | Ccl8 | Ccl8 | NM_021443 |
| 122 | scl0094185.2_20-S | tumor necrosis factor receptor superfamily, member 21 | Tnfrsf21 | Tnfrsf21 | NM_178589 |
| 123 | scl017921.2_30-S | myosin VIIa | Myo7a | Myo7a | NM_008663 |
| 124 | scl47523.9_497-S | glycerol-3-phosphate dehydrogenase 1 (soluble) | Gpd1 | Gpd1 | NM_010271 |
| 125 | scl33167.10_5-S | cDNA sequence BC021891 | BC021891 | BC021891 | NM_145608 |
| 126 | scl54609.9_173-S | proteolipid protein 1 | Plp1 | Plp1 | NM_011123 |
| 127 | scl21271.30_25-S | mannose receptor, C type 1 | Mrc1 | Mrc1 | NM_008625 |
| 128 | scl43993.5.1_29-S | sushi domain containing 3 | Susd3 | Susd3 | NM_025491 |
| 129 | scl0003156.1_0-S | WAP four-disulfide core domain 2 | Wfdc2 | Wfdc2 | NM_026323 |
| 130 | scl077717.2_28-S | hypothetical protein LOC77717 | 6030408B16Rik |  | XM_358478 |
| 131 | scl21235.24_380-S |  |  |  | NM_029895 |
| 132 | scl46716.10.1_32-S | elastase 1, pancreatic | Ela1 | Ela1 | NM_033612 |
| 133 | scl18127.10.1_92-S | glutathione S-transferase, alpha 3 | Gsta3 | Gsta3 | NM_010356 |
| 134 | scl078938.5_60-S | F-box only protein 34 | Fbxo34 | Fbxo34 | NM_030236 |
| 135 | scl0013078.2_259-S | cytochrome P450, family 1, subfamily b, polypeptide 1 | Cyp1b1 | Cyp1b1 | NM_009994 |
| 136 | scl21571.6.1_18-S | myozenin 2 | Myoz2 | Myoz2 | NM_021503 |
| 137 | scl012268.2_49-S | complement component 4 (within H-2S) | C4 | C4b | NM_009780 |
| 138 | scl0001167.1_16-S | LIM and cysteine-rich domains 1 | Lmcd1 | Lmcd1 | NM_144799 |
| 139 | scl076071.13_258-S | gamma-aminobutyric acid (GABA-B) receptor binding protein | Gababrbp | Jakmip1 | NM_178394 |
| 140 | scl072736.8_85-S | thioredoxin domain containing 1 | Txndc1 | Txndc1 | NM_028339 |
| 141 | scl28557.1_516-S | brain stress early protein Gbi isoform 1 | Srgap3 |  | NM_153070 |
| 142 | scl0002358.1_1625-S | tripartite motif protein 9 | Trim9 | Trim9 | AK129109 |
| 143 | scl46158.7.1_95-S | cholinergic receptor, nicotinic, alpha polypeptide 2 (neuronal) | Chrna2 | Chrna2 | NM_144803 |
| 144 | scl011723.1_3-S | amylase 2, pancreatic | Amy1 | Amy2 | NM_009669 |
| 145 | scl0223649.1_255-S | nuclear receptor binding protein 2 | BC011468 | Nrbp2 | NM_144847 |
| 146 | scl36679.7.1_96-S | glutathione S-transferase, alpha 4 | Gsta4 | Gsta4 | NM_010357 |
| 147 | scl38423.9.1_9-S | tetraspanin 8 | Tm4sf3 | Tspan8 | NM_146010 |
| 148 | scl38511.3.1_54-S | decorin | Dcn | Dcn | NM_007833 |
| 149 | scl027877.2_206-S |  |  |  |  |
| 150 | GI_51921354-S |  |  |  |  |
| 151 | scl27703.26_20-S | platelet derived growth factor receptor, alpha polypeptide | Pdgfra | Pdgfra | NM_011058 |
| 152 | scl0001887.1_26-S | phospholipase A1 member A | Pla1a | Pla1a | NM_134102 |
| 153 | scl40921.6_29-S | insulin-like growth factor binding protein 4 | Igfbp4 | Igfbp4 | NM_010517 |
| 154 | scl17872.1_157-S |  |  |  |  |
| 155 | scl36816.23_421-S | neighbor of Punc e11 protein | Nope | Nope | NM_020043 |
| 156 | scl42725.12.1_79-S | phospholipase D family, member 4 | AI132321 | Pld4 | NM_178911 |
| 157 | scl0076898.1_65-S | beta-1,3-glucuronyltransferase 1 (glucuronosyltransferase P) | B3gat1 | B3gat1 | NM_029792 |
| 158 | scl17296.11_403-S | vesicle-associated membrane protein 4 | Vamp4 | Vamp4 | NM_016796 |
| 159 | scl44779.2.1_92-S | nucleoredoxin | 4930519N16Rik | 4930519N16Rik | NM_029173 |
| 160 | GI_85702287-S |  |  |  |  |
| 161 | scl17155.4.1_166-S | hypothetical protein LOC269152 | 4832420M10 | Kif26b | NM_177757 |
| 162 | scl32804.19.1_0-S | dermokine alpha | Dmkn; SK30; SK89; cI-36; AW561900; C130074A08 | Dmkn | NM_172899 |
| 163 | scl39086.7.1_160-S | vanin 3 | Vnn3 | Vnn3 | NM_011979 |
| 164 | scl52691.13_82-S | phosphoserine aminotransferase 1 | Psat1 |  | XM_129211 |
| 165 | scl49626.40.1_3-S | xanthine dehydrogenase | Xdh | Xdh | NM_011723 |
| 166 | scl016000.2_30-S | insulin-like growth factor 1 isoform 1 | Igf1 | Igf1 | NM_010512 |
| 167 | GI_38077621-S |  |  |  | XM_356826 |
| 168 | scl0054132.2_227-S | carboxyl terminal LIM domain protein 1 | Pdlim1 | Pdlim1 | NM_016861 |
| 169 | scl2370.1.1_197-S |  |  |  |  |
| 170 | scl023792.26_1-S | a disintegrin and metallopeptidase domain 23 preproprotein | Adam23 | Adam23 | NM_011780 |
| 171 | scl37681.14_50-S | solute carrier family 41, member 2 | Slc41a2 | Slc41a2 | NM_177388 |
| 172 | scl0384061.7_167-S | fibronectin type III domain containing 5 | Fndc5 | Fndc5 | NM_027402 |
| 173 | scl0072098.1_29-S | transmembrane protein 68 | 2010300G19Rik | Tmem68 | NM_028097 |
| 174 | scl015483.1_7-S | hydroxysteroid 11-beta dehydrogenase 1 | Hsd11b1 | Hsd11b1 | NM_008288 |
| 175 | scl19917.13_586-S | matrix metalloproteinase 9 | Mmp9 | Mmp9 | NM_013599 |
| 176 | scl25132.8_177-S | calreticulin 4 | 4933403L16Rik |  | XM_205476 |
| 177 | scl0106068.1_320-S | hypothetical protein LOC106068 isoform 1 | 9330175B01Rik |  | XM_128267 |
| 178 | scl41403.1.1_37-S |  |  |  |  |
| 179 | scl19431.33.1_37-S | GTPase activating RANGAP domain-like 3 | Garnl3 | Garnl3 | NM_178888 |
| 180 | ri|1500001L03|ZX00050C19|AK005096|1469-S | fibronectin type III domain containing 5 | Fndc5 | Fndc5 | AK005096 |
| 181 | scl47422.17_72-S | oncostatin M receptor | Osmr | Osmr | NM_011019 |
| 182 | scl45563.11.1_75-S | solute carrier family 7 (cationic amino acid transporter, y+ system), member 8 | Slc7a8 | Slc7a8 | NM_016972 |
| 183 | scl0004049.1_686-S | regulator of G-protein signalling 12 | Rgs12 | Rgs12 | NM_173402 |
| 184 | scl16390.7_3-S | v-ral simian leukemia viral oncogene homolog B (ras related) | Ralb | Ralb | NM_022327 |
| 185 | scl000857.1_11-S | hydroxysteroid 11-beta dehydrogenase 1 | Hsd11b1 | Hsd11b1 | NM_008288 |
| 186 | scl012521.1_26-S | kangai 1 (suppression of tumorigenicity 6, prostate) | Kai1 | Cd82 | NM_007656 |
| 187 | scl28822.22_22-S | catenin (cadherin associated protein), alpha 2 isoform 2 | Catna2 | Ctnna2 | NM_009819 |
| 188 | scl42954.11_322-S | hypothetical protein LOC217721 | BC011209 | Flvcr2 | NM_145447 |
| 189 | scl35308.7.1_3-S | integral membrane protein TMIE | Tmie | Tmie | NM_146260 |
| 190 | scl0214639.2_15-S | testin | 4930486L24Rik | 4930486L24Rik | NM_178098 |
| 191 | scl49773.2_28-S | leucine-rich alpha-2-glycoprotein | Lrg1 | Lrg1 | NM_029796 |
| 192 | scl076630.10_6-S | AMSH-family protein | 1700095N21Rik | Stambpl1 | NM_029682 |
| 193 | scl4670.1.1_55-S |  |  |  |  |
| 194 | scl16190.6_258-S | regulator of G-protein signaling 2 | Rgs2 | Rgs2 | NM_009061 |
| 195 | ri|A230050B20|PX00128B15|AK038610|974-S | hypothetical protein LOC414083 | 8030463A06Rik | 8030463A06Rik | AK038610 |
| 196 | scl098952.11_256-S | early estrogen-induced gene 1 protein | C230093N12Rik | C230093N12Rik | NM_153560 |
| 197 | GI_38081895-S |  |  |  | XM_124621 |
| 198 | scl0013384.1_233-S | Mpp3 membrane protein, palmitoylated 3 | Mpp3 | Mpp3 | NM_007863 |
| 199 | scl38099.61.1_34-S | laminin, alpha 2 | Lama2 | Lama2 | NM_008481 |
| 200 | scl0015446.2_268-S | hydroxyprostaglandin dehydrogenase 15 (NAD) | Hpgd | Hpgd | NM_008278 |
| 201 | scl00241556.2_5-S | tetraspanin 18 | 6720430O15 | Tspan18 | NM_183180 |
| 202 | scl066259.1_113-S |  |  |  | NM_025451 |
| 203 | scl016779.32_71-S | laminin, beta 2 | Lamb2 | Lamb2 | NM_008483 |
| 204 | scl17938.36.1_1-S | aldehyde oxidase structural homolog 2 | Aox3 | Aox3 | NM_023617 |
| 205 | scl49330.22.4_270-S | chordin | Chrd | Chrd | NM_009893 |
| 206 | scl0003519.1_175-S | neural cell adhesion molecule 1 | Ncam1 | Ncam1 | X06328 |
| 207 | scl54811.82_56-S | dystrophin, muscular dystrophy | Dmd | Dmd | NM_007868 |
| 208 | GI_38081359-S |  |  |  | XM_355658 |
| 209 | scl0209966.1_129-S | piggyBac transposable element derived 5 | Pgbd5 | Pgbd5 | NM_171824 |
| 210 | ri|8030463A06|PX00103L03|AK033210|2619-S | hypothetical protein LOC414083 | 8030463A06Rik | 8030463A06Rik | AK033210 |
| 211 | scl000293.1_16-S | claudin 10 isoform b | Cldn10 | Cldn10 | NM_021386 |
| 212 | scl39634.14_208-S | plexin domain containing 1 | Plxdc1 | Plxdc1 | NM_028199 |
| 213 | GI_6755419-S |  |  |  |  |
| 214 | scl0002507.1_236-S | fibulin 1 | Fbln1 | Fbln1 | AK083573 |
| 215 | ri|4632409D22|PX00637I12|AK076278|2778-S |  | AW743884 | Col12a1 | AK076278 |
| 216 | scl0019735.2_283-S | regulator of G-protein signaling 2 | Rgs2 | Rgs2 | NM_009061 |
| 217 | scl27070.12.1_10-S | cDNA sequence BC004044 | BC004044 | BC004044 | NM_030565 |
| 218 | scl26695.10.1_5-S | carboxypeptidase Z | Cpz | Cpz | NM_153107 |
| 219 | scl34312.7.1_89-S | chymotrypsinogen B1 | Ctrb1 | Ctrb1 | NM_025583 |
| 220 | scl53819.3_141-S | brain expressed X-linked 2 | Bex2 | Bex2 | NM_009749 |
| 221 | scl54605.3_321-S | SMAD-interacting zinc finger protein 2 | 1500031H04Rik | Zcchc18 | NM_025893 |
| 222 | scl069598.1_26-S | pyridoxal (pyridoxine, vitamin B6) kinase | Pdxk |  | XM_488563 |
| 223 | scl00223473.2_241-S | NIPA-like domain containing 2 | 9330161F08Rik | Npal2 | NM_145469 |
| 224 | GI_85702180-S |  |  |  |  |
| 225 | scl42524.16_317-S | scinderin | Scin | Scin | NM_009132 |
| 226 | scl0243362.1_178-S | serologically defined colon cancer antigen 13 | Stard13 | Stard13 | NM_146258 |
| 227 | scl38708.12_196-S | paralemmin | Palm | Palm | NM_023128 |
| 228 | scl44826.11.1_30-S | guanosine monophosphate reductase | Gmpr | Gmpr | NM_025508 |
| 229 | scl18632.11_218-S | Ras association (RalGDS/AF-6) domain family 2 | Rassf2 | Rassf2 | NM_175445 |
| 230 | scl45410.6_648-S | scavenger receptor class A, member 3 | Scara3 | Scara3 | NM_172604 |
| 231 | scl24849.9.1_206-S | similar to solute carrier family 30, member 2 | Slc30a2 |  | XM_131731 |
| 232 | scl00235534.1_198-S | acid phosphatase-like 2 | Acpl2 | Acpl2 | NM_153420 |
| 233 | scl36159.7.1_126-S | angiopoietin-related protein 5 | Angptl6 | Angptl6 | NM_145154 |
| 234 | scl32598.21.1_0-S | ATPase, class V, type 10A | Atp10a | Atp10a | NM_009728 |
| 235 | scl16572.10_105-S | serine (or cysteine) proteinase inhibitor, clade E, member 2 | Serpine2 | Serpine2 | NM_009255 |
| 236 | scl22481.4_67-S | endothelial differentiation, lysophosphatidic acid G-protein-coupled receptor 7 | Edg7 | Edg7 | NM_022983 |
| 237 | scl33196.2.1635_134-S | ras homolog gene family, member U | Rhou | Rhou | NM_133955 |
| 238 | ri|1110018K11|R000014D02|AK003784|499-S | homeobox only domain | Hod |  | AK003784 |
| 239 | scl00237159.1_186-S |  |  |  | NM_146237 |
| 240 | scl40807.26_6-S | angiotensin I converting enzyme (peptidyl-dipeptidase A) 1 isoform 2 | Ace | Ace | NM_009598 |
| 241 | scl0020661.1_145-S | sortilin 1 | Sort1 | Sort1 | NM_019972 |
| 242 | scl00224022.2_173-S | solute carrier family 7 (cationic amino acid transporter, y+ system), member 4 | Slc7a4 | Slc7a4 | NM_144852 |
| 243 | scl31916.12.1_47-S | scavenger receptor cysteine-rich type 1 protein CD163c-alpha | E430002D04Rik | E430002D04Rik | NM_172909 |
| 244 | scl50824.6.23_33-S | histocompatibility 2, class II antigen E beta | H2-Eb1 | H2-Eb1 | NM_010382 |
| 245 | scl39611.13_705-S | C-terminal tensin-like | 9930017A07Rik | Tns4 | NM_172564 |
| 246 | scl0054613.2_126-S | alpha2,3-sialyltransferase VI | Siat10 | St3gal6 | NM_018784 |
| 247 | scl017069.4_4-S | lymphocyte antigen 6 complex, locus E | Ly6e | Ly6e | NM_008529 |
| 248 | scl19180.25_455-S | sodium channel, voltage-gated, type VII, alpha polypeptide | Scn7a | Scn7a | NM_009135 |
| 249 | ri|2310058A03|ZX00040B05|AK009971|906-S | WAP four-disulfide core domain 1 | ps20; 2310058A03Rik | Wfdc1 | AK009971 |
| 250 | scl068792.10_256-S | sushi-repeat containing protein | Srpx2 | Srpx2 | NM_026838 |
| 251 | GI_40254606-A |  |  |  |  |
| 252 | GI_31982555-S | interferon activated gene 205 | Ifi202b | Ifi205 | NM_172648 |
| 253 | ri|G430069A15|PH00001D14|AK090036|1282-S | FK506 binding protein 6 | Fkbp6 | Fkbp6 | AK090036 |
| 254 | scl27966.3.1_97-S | transcription factor 23 | Tcf23 | Tcf23 | NM_053085 |
| 255 | scl16121.8.1_163-S | hypothetical protein XP_148974 isoform 1 | BC034090 | BC034090 | XM_148974 |
| 256 | scl0103250.1_3-S |  |  |  |  |
| 257 | scl37806.7_98-S | matrix metalloproteinase 11 | Mmp11 | Mmp11 | NM_008606 |
| 258 | scl31674.3.1_6-S | apolipoprotein C-I | Apoc1 | Apoc1 | NM_007469 |
| 259 | scl31107.6.1_14-S | hypothetical protein LOC210321 | BC048679 | BC048679 | NM_183143 |
| 260 | scl30224.10.1_23-S | aldo-keto reductase family 1, member B7 | Akr1b7 | Akr1b7 | NM_009731 |
| 261 | ri|4833436O22|PX00028P19|AK029441|2683-S |  |  | 1200016E24Rik | AK029441 |
| 262 | scl25131.24_224-S | epidermal growth factor receptor pathway substrate 15 | Eps15 | Eps15 | NM_007943 |
| 263 | scl47060.10.1_13-S | nicotinate phosphoribosyltransferase domain containing 1 | 9130210N20Rik | Naprt1 | NM_172607 |
| 264 | scl0170755.15_107-S | serum/glucocorticoid regulated kinase 3 | Cisk; 2510015P22Rik; A330005P07Rik | Sgk3 | NM_177547 |
| 265 | scl26598.18_80-S | G protein-coupled receptor 125 isoform 1 | Gpr125 | Gpr125 | XM_132089 |
| 266 | scl20308.8.1_3-S | hypothetical protein LOC70370 | 1600015H20Rik | 1600015H20Rik | NM_024237 |
| 267 | scl33795.5_730-S | microfibrillar-associated protein 3-like | Mfap3l | Mfap3l | NM_027756 |
| 268 | ri|4930432N22|PX00031K13|AK076756|1763-S | zinc ring finger protein 1 | Znrf1 | Znrf1 | AK076756 |
| 269 | scl0105278.8_30-S | cell cycle related kinase | Ccrk | Ccrk | NM_053180 |
| 270 | scl0019041.2_232-S |  |  |  | XM_148334 |
| 271 | scl28339.6.1_65-S | macrophage antigen h | 4933425B16Rik | 4933425B16Rik | XM_132881 |
| 272 | scl55045.9_124-S | tetraspanin 7 | Tm4sf2 | Tspan7 | NM_019634 |
| 273 | scl38757.5.1_81-S | Nur77 downstream protein 2 | Ndg2 |  | NM_175329 |
| 274 | scl38695.24.1_79-S | minor histocompatibility antigen HA-1 | 6330406L22Rik | Hmha1 | NM_027521 |
| 275 | scl49315.8.1_6-S | alpha-2-HS-glycoprotein | Ahsg | Ahsg | NM_013465 |
| 276 | scl36891.2_185-S | cytochrome P450, family 11, subfamily a, polypeptide 1 | Cyp11a1 | Cyp11a1 | NM_019779 |
| 277 | scl48036.13_340-S | ank protein | Ank | Ank | NM_020332 |
| 278 | scl17480.9.1_6-S | NUAK family, SNF1-like kinase, 2 | 1200013B22Rik | Nuak2 | NM_028778 |
| 279 | scl31526.16.1_20-S | amyloid precursor-like protein 1 | Aplp1 | Aplp1 | NM_007467 |
| 280 | scl38989.5.1_64-S | hypothetical protein LOC327747 | 9030224M15Rik | 9030224M15Rik | NM_177793 |
| 281 | scl000900.1_4-S | serum/glucocorticoid regulated kinase 3 | Cisk; 2510015P22Rik; A330005P07Rik | Sgk3 | NM_177547 |
| 282 | scl23487.8.3034_55-S | hexose-6-phosphate dehydrogenase (glucose 1-dehydrogenase) | H6pd | H6pd | NM_173371 |
| 283 | scl0001013.1_0-S | B-cell leukemia/lymphoma 2 isoform 1 | Bcl2 | Bcl2 | NM_177410 |
| 284 | scl50975.2_304-S |  |  |  |  |
| 285 | scl014862.2_242-S | glutathione S-transferase, mu 1 | Gstm1 | Gstm1 | NM_010358 |
| 286 | scl0110542.11_226-S | anti-Mullerian hormone type 2 receptor | Amhr2 | Amhr2 | NM_144547 |
| 287 | scl49983.22_274-S | discoidin domain receptor family, member 1 | Ddr1 | Ddr1 | NM_007584 |
| 288 | scl00319202.1_96-S |  |  |  | XM_489305 |
| 289 | scl36356.8_626-S | CTD (carboxy-terminal domain, RNA polymerase II, polypeptide A) small phosphatase-like | Ctdspl | Ctdspl | NM_133710 |
| 290 | scl27391.19_438-S | acetyl-Coenzyme A carboxylase beta | Acacb | Acacb | NM_133904 |
| 291 | scl25526.10_573-S | hypothetical protein LOC230085 | N28178 | N28178 | NM_172690 |
| 292 | scl0268822.14_46-S | aarF domain containing kinase 5 | Adck5 | Adck5 | NM_172960 |
| 293 | scl17488.7_66-S | prostate cancer associated protein 6 | Pcanap6 | Slc45a3 | NM_145977 |
| 294 | scl48380.16.1_85-S | G protein-coupled receptor 128 | Gpr128 | Gpr128 | NM_172825 |
| 295 | scl52314.6.1_5-S | peroxiredoxin 3 | Prdx3 | Prdx3 | NM_007452 |
| 296 | scl18131.5_291-S | progestin and adipoQ receptor family member VIII | 1700019B16Rik | Paqr8 | NM_028829 |
| 297 | scl0003759.1_6-S | H2A histone family, member Y | H2afy | H2afy | BC006955 |
| 298 | GI_45383917-A |  |  |  |  |
| 299 | scl066953.9_91-S | cell division cycle associated 7 | Cdca7 | Cdca7 | NM_025866 |
| 300 | scl43045.31_238-S | pleckstrin homology domain containing, family H (with MyTH4 domain) member 1 isoform 1 | Plekhh1 | Plekhh1 | XM_126961 |
| 301 | scl22011.14.1179_292-S | hypothetical protein LOC229474 | 6330505N24Rik |  | XM_130991 |
| 302 | scl51657.9.1_30-S | abhydrolase domain containing 3 | Abhd3 | Abhd3 | NM_134130 |
| 303 | scl42101.2_23-S | serine (or cysteine) proteinase inhibitor, clade A, member 3C | Serpina3c | Serpina3c | NM_008458 |
| 304 | scl013121.10_1-S | cytochrome P450, family 51 | Cyp51 | Cyp51 | NM_020010 |
| 305 | scl29827.46.1_90-S | dysferlin | Dysf | Dysf | NM_021469 |
| 306 | scl0014732.2_168-S | glycerol-3-phosphate acyltransferase, mitochondrial | Gpam | Gpam | NM_008149 |
| 307 | scl52091.9.1_29-S |  |  |  | XM_488847 |
| 308 | scl076263.8_129-S | glutathione S-transferase class kappa | Gstk1 | Gstk1 | NM_029555 |
| 309 | scl41590.4.1_265-S | hypothetical protein LOC97775 | D930048N14Rik |  | NM_175289 |
| 310 | scl0022218.1_0-S | SMT3 suppressor of mif two 3 homolog 1 | Sumo1 | Sumo1 | NM_009460 |
| 311 | scl32668.4.1_181-S | D site albumin promoter binding protein | Dbp | Dbp | NM_016974 |
| 312 | GI_38076230-S |  |  |  | XM_355397 |
| 313 | scl16675.14_10-S | isocitrate dehydrogenase 1 (NADP+), soluble | Idh1 | Idh1 | NM_010497 |
| 314 | scl32676.9.1_1-S | pleckstrin homology domain-containing, family A member 4 | Plekha4 | Plekha4 | NM_148927 |
| 315 | scl0056473.2_148-S | fatty acid desaturase 2 | Fads2 | Fads2 | NM_019699 |
| 316 | scl00319158.2_61-S | histone 1, H4i | Hist1h4i | Hist1h4i | NM_175656 |
| 317 | scl47204.11.1_33-S | exostosin 1 | Ext1 | Ext1 | NM_010162 |
| 318 | scl017751.1_8-S | metallothionein 3 | Mt3 | Mt3 | NM_013603 |
| 319 | GI_38080559-S |  |  |  | XM_358790 |
| 320 | scl33411.5.1_30-S | hydroxysteroid 11-beta dehydrogenase 2 | Hsd11b2 | Hsd11b2 | NM_008289 |
| 321 | scl00170459.2_123-S | StAR-related lipid transfer (START) domain containing 4 | Stard4 | Stard4 | NM_133774 |
| 322 | scl31222.7.115_17-S | desmuslin isoform L | Dmn | Dmn | NM_207663 |
| 323 | scl51725.12_3-S | Golli-mbp isoform 2 | Mbp | Mbp | NM_010777 |
| 324 | scl0002655.1_13-S | tropomyosin 2, beta | Tpm2 | Tpm2 | NM_009416 |
| 325 | scl19685.22.1_10-S | inter-alpha trypsin inhibitor, heavy chain 2 | Itih2 | Itih2 | NM_010582 |
| 326 | scl011668.12_94-S | aldehyde dehydrogenase family 1, subfamily A1 | Aldh1a1 | Aldh1a1 | NM_013467 |
| 327 | scl45684.12_151-S | tetraspanin 14 | Tm4sf14 | Tspan14 | NM_145928 |
| 328 | scl23013.4.1_25-S | cellular retinoic acid binding protein II | Crabp2 | Crabp2 | NM_007759 |
| 329 | ri|1200006J22|R000008J07|AK004616|1702-S | solute carrier organic anion transporter family, member 2a1 | Slco2a1 | Slco2a1 | AK004616 |
| 330 | scl16555.3_197-S | insulin receptor substrate 1 | Irs1 | Irs1 | NM_010570 |
| 331 | scl22560.5_561-S | DNA-damage-inducible transcript 4-like | Ddit4l | Ddit4l | NM_030143 |
| 332 | scl0100669.2_129-S |  |  |  |  |
| 333 | scl0210356.1_160-S | Nck-associated protein 5 | E030049G20Rik |  | XM_358333 |
| 334 | scl020969.4_58-S | syndecan 1 | Sdc1 | Sdc1 | NM_011519 |
| 335 | scl0020230.2_317-S | special AT-rich sequence binding protein 1 | Satb1 | Satb1 | NM_009122 |
| 336 | scl076257.1_48-S | N system amino acids transporter NAT-1 | Slc38a3 | Slc38a3 | NM_023805 |
| 337 | scl0003469.1_9-S | acyl-Coenzyme A dehydrogenase family, member 8 | Acad8 | Acad8 | NM_025862 |
| 338 | scl0207683.7_143-S | immunoglobulin superfamily, member 11 | Igsf11 | Igsf11 | NM_170599 |
| 339 | ri|D130059O18|PX00185A22|AK051608|1931-S | arginine/serine-rich splicing factor 6 | Sfrs6 | Sfrs6 | AK051608 |
| 340 | scl022249.50_191-S | unc-13 homolog B | Unc13b |  | NM_021468 |
| 341 | scl020778.1_265-S | scavenger receptor class B, member 1 | Scarb1 | Scarb1 | NM_016741 |
| 342 | scl52737.9.1_36-S | membrane-spanning 4-domains, subfamily A, member 10 | Ms4a10 | Ms4a10 | NM_023529 |
| 343 | scl0057266.1_102-S | kidney-expressed chemokine CXC | Cxcl14 | Cxcl14 | NM_019568 |
| 344 | scl41542.4_321-S | GM2 ganglioside activator protein | Gm2a | Gm2a | NM_010299 |
| 345 | GI_41235740-S | hypothetical protein LOC381110 | AW061290 | AW061290 | NM_201361 |
| 346 | scl00226778.2_143-S | MAP/microtubule affinity-regulating kinase 1 | Mark1 | Mark1 | NM_145515 |
| 347 | scl0004054.1_5-S | scavenger receptor class B, member 1 | Scarb1 | Scarb1 | NM_016741 |
| 348 | scl0002596.1_12-S | diaphorase 1 | Dia1 | Cyb5r3 | NM_029787 |
| 349 | scl44517.9_405-S | lipoma HMGIC fusion partner-like 2 | Lhfpl2 | Lhfpl2 | NM_172589 |
| 350 | scl000928.1_86-S | heat shock protein 1 (chaperonin) | Hspd1 | Hspd1 | NM_010477 |
| 351 | scl0013640.1_290-S | ephrin A5 isoform 2 | Efna5 | Efna5 | NM_207654 |
| 352 | scl068157.2_24-S |  |  |  |  |
| 353 | scl38453.7.1_27-S | cysteine and glycine-rich protein 2 | Csrp2 | Csrp2 | NM_007792 |
| 354 | scl52812.25.1_7-S | pyruvate carboxylase | Pcx | Pcx | NM_008797 |
| 355 | scl073737.1_220-S | hypothetical protein LOC73737 | 1110008P14Rik | 1110008P14Rik | NM_198001 |
| 356 | scl063955.10_154-S | Cdk5 and Abl enzyme substrate 1 | Cables1 | Cables1 | NM_022021 |
| 357 | scl34499.5.1_0-S | Iroquois related homeobox 3 | Irx3 | Irx3 | NM_008393 |
| 358 | ri|4930426D05|PX00030N19|AK015205|1620-S |  |  |  | AK015205 |
| 359 | scl33658.4_26-S | RASD family, member 2 | Rasd2 | Rasd2 | XM_204287 |
| 360 | scl0003060.1_2-S | adenosine deaminase | Ada | Ada | NM_007398 |
| 361 | scl0003547.1_6-S |  |  |  |  |
| 362 | scl0003582.1_66-S | acyl-Coenzyme A dehydrogenase family, member 8 | Acad8 | Acad8 | NM_025862 |
| 363 | scl22134.5_174-S | profilin 2 | Pfn2 | Pfn2 | NM_019410 |
| 364 | scl0002952.1_136-S | hephaestin isoform 1 | Heph | Heph | NM_010417 |
| 365 | scl17826.1.31_58-S | insulin-like growth factor binding protein 2 | Igfbp2 | Igfbp2 | NM_008342 |
| 366 | GI_20892928-S |  |  |  | XM_147218 |
| 367 | scl27701.21_88-S | c-kit | Kit | Kit | NM_021099 |
| 368 | ri|A630063N04|PX00147I11|AK042147|923-S | prolactin receptor | Prlr | Prlr | AK042147 |
| 369 | scl0015484.2_48-S | hydroxysteroid 11-beta dehydrogenase 2 | Hsd11b2 | Hsd11b2 | NM_008289 |
| 370 | scl18988.12.1_11-S | glycosyltransferase-like 1B | Gyltl1b | Gyltl1b | NM_172670 |
| 371 | scl18196.2_265-S | SRY-box containing gene 18 | Sox18 | Sox18 | NM_009236 |
| 372 | scl20117.4.1_53-S | hypothetical protein LOC319579 | 9230107O10Rik | 9230107O10Rik | NM_176950 |
| 373 | scl30187.2.1_182-S | hypothetical protein LOC66117 | 1110001J03Rik | 1110001J03Rik | NM_025363 |
| 374 | scl37275.1_87-S | sestrin 3 | Sesn3 | Sesn3 | NM_030261 |
| 375 | scl53077.5.8_20-S | glutathione S-transferase omega 1 | Gsto1 | Gsto1 | NM_010362 |
| 376 | scl27547.3.1_4-S | bone morphogenetic protein 3 | Bmp3 | Bmp3 | NM_173404 |
| 377 | scl53247.20_415-S | very low density lipoprotein receptor | Vldlr | Vldlr | NM_013703 |
| 378 | scl53459.3.1_19-S | ras homolog D | Rhod | Rhod | NM_007485 |
| 379 | scl50455.10_283-S | hypothetical protein LOC106522 | AW548124 |  | XM_128778 |
| 380 | scl014528.1_141-S | GTP cyclohydrolase 1 | Gch1 | Gch1 | NM_008102 |
| 381 | scl0015926.2_189-S | isocitrate dehydrogenase 1 (NADP+), soluble | Idh1 | Idh1 | NM_010497 |
| 382 | scl00319161.1_8-S | histone 1, H4m | Hist1h4m | Hist1h4m | NM_175657 |
| 383 | scl0240168.18_64-S | RAS, guanyl releasing protein 3 | Rasgrp3 | Rasgrp3 | NM_207246 |
| 384 | scl0353509.1_0-S | chemokine-like factor super family 1 | Cklfsf2a | Cmtm1 | NM_181990 |
| 385 | scl011808.3_227-S | apolipoprotein A-IV | Apoa4 | Apoa4 | NM_007468 |
| 386 | scl020379.6_202-S | secreted frizzled-related sequence protein 4 | Sfrp4 | Sfrp4 | NM_016687 |
| 387 | scl077574.1_0-S | hypothetical protein LOC77574 | 3321401G04Rik |  | XM_133096 |
| 388 | scl29611.11.1_50-S | peroxisome proliferator activated receptor gamma | Pparg | Pparg | NM_011146 |
| 389 | scl32818.7.1_108-S | hypothetical protein LOC233066 | AI428936 | AI428936 | NM_153577 |
| 390 | scl070083.1_37-S | meteorin | Metrn | Metrn | NM_133719 |
| 391 | scl37803.16.1_107-S | solute carrier family 5 member 4b | Slc5a4b | Slc5a4b | NM_023219 |
| 392 | scl30359.11_166-S | testis derived transcript isoform 1 | Tes | Tes | NM_011570 |
| 393 | scl0056458.2_48-S | forkhead box O1a | Foxo1 | Foxo1 | NM_019739 |
| 394 | ri|D130083G05|PX00186P02|AK084071|1297-S | hypothetical protein LOC545015 | 2610042L04Rik | ENSMUSG00000057445 | AK084071 |
| 395 | ri|A630086H07|PX00147H03|AK080384|970-S |  | A630053O10 | Iqgap2 | AK080384 |
| 396 | scl36687.1_397-S | hypothetical protein LOC320711 | 9830147P19Rik |  | NM_177238 |
| 397 | scl16606.3_174-S | Indian hedgehog | Ihh | Ihh | NM_010544 |
| 398 | scl020715.6_281-S |  |  |  | XM_354694 |
| 399 | scl41049.3_319-S | hypothetical protein LOC67888 | 1810057C19Rik | Tmem100 | NM_026433 |
| 400 | scl32679.14.1_2-S | tubby-like protein 2 | Tulp2 | Tulp2 | NM_008807 |
| 401 | scl54768.23.4_19-S | hephaestin isoform 1 | Heph | Heph | NM_010417 |
| 402 | GI_38080156-S | hypothetical protein LOC239691 | AU021092 |  | XM_358755 |
| 403 | scl0218121.14_31-S | O-acyltransferase (membrane bound) domain containing 1 | Oact1 | Mboat1 | NM_153546 |
| 404 | scl35042.9.1_7-S | angiopoietin 2 | Angpt2 | Angpt2 | NM_007426 |
| 405 | scl00268977.2_205-S | latent transforming growth factor beta binding protein 1 isoform a | Ltbp1 | Ltbp1 | NM_019919 |
| 406 | ri|A530075A22|PX00142K10|AK080168|541-S |  | ENSMUSG00000055015 | ENSMUSG00000055015 | AK080168 |
| 407 | scl32603.25.9_1-S | pink-eyed dilution protein | p | p | NM_021879 |
| 408 | scl43239.32_393-S |  |  |  | XM_147660 |
| 409 | scl45280.1.3_130-S |  |  |  |  |
| 410 | scl0022359.2_126-S | very low density lipoprotein receptor | Vldlr | Vldlr | NM_013703 |
| 411 | scl41372.14.1_133-S | potassium voltage-gated channel, shaker-related subfamily, beta member 3 | Kcnab3 | Kcnab3 | NM_010599 |
| 412 | scl45193.8_18-S | endothelin receptor type B | Ednrb | Ednrb | NM_007904 |
| 413 | scl45683.9_474-S | hypothetical protein LOC70564 | 5730469M10Rik | 5730469M10Rik | NM_027464 |
| 414 | scl15160.1.1_303-S | MEGF10 protein | 3000002B06Rik | Megf10 | NM_001001979 |
| 415 | scl27301.8_458-S | T-box 3 protein isoform 1 | Tbx3 | Tbx3 | NM_011535 |
| 416 | 18S_rRNA_X00686_523-S | phosphatidylinositol glycan, class T | CGI-06; 4930534E15Rik | Pigt | NM_133779 |
| 417 | scl55015.6.1_13-S | tissue inhibitor of metalloproteinase 1 | Timp1 | Timp1 | NM_011593 |
| 418 | scl026557.1_7-S | homer homolog 2 | Homer2 |  | XM_133550 |
| 419 | scl30959.7.1_15-S | folate receptor 1 (adult) | Folr1 | Folr1 | NM_008034 |
| 420 | scl0019419.1_154-S | RAS guanyl releasing protein 1 | Rasgrp1 | Rasgrp1 | NM_011246 |
| 421 | scl42787.13.1_20-S | GTL2, imprinted maternally expressed untranslated | Gtl2 |  | NM_144513 |
| 422 | scl0022379.1_143-S | formin-like 3 protein | Fmnl3 | Fmnl3 | NM_011711 |
| 423 | scl52399.8.1_189-S | cytochrome P450, family 17, subfamily a, polypeptide 1 | Cyp17a1 | Cyp17a1 | NM_007809 |
| 424 | scl011754.4_303-S | amine oxidase, copper containing 3 | Aoc3 | Aoc3 | NM_009675 |
| 425 | scl23489.7_25-S | SPRY domain-containing SOCS box protein SSB-1 | 4930422J18Rik | Spsb1 | NM_029035 |
| 426 | scl35377.11.1_104-S | HemK methyltransferase family member 1 | Hemk1 | Hemk1 | NM_133984 |
| 427 | scl35842.10.1_195-S | cytochrome P450, family 19, subfamily a, polypeptide 1 | Cyp19a1 | Cyp19a1 | NM_007810 |
| 428 | scl41866.19_12-S | AE binding protein 1 | Aebp1 | Aebp1 | NM_009636 |
| 429 | scl15839.11.1_64-S | epoxide hydrolase 1, microsomal | Ephx1 | Ephx1 | NM_010145 |
| 430 | scl51859.1.3752_6-S |  |  |  |  |
| 431 | scl21825.13.80_16-S | carbonic anhydrase 14 | Car14 | Car14 | NM_011797 |
| 432 | scl28020.17.1_9-S | ARP3 actin-related protein 3 homolog B | Arp3b | Actr3b | NM_001004365 |
| 433 | scl35940.9_671-S | transmembrane protein 25 | Tmem25 | Tmem25 | NM_027865 |
| 434 | scl51099.14_176-S | secreted modular calcium-binding protein 2 | Smoc2 | Smoc2 | NM_022315 |
| 435 | scl0001889.1_55-S | beta-site APP-cleaving enzyme 2 | Bace2 | Bace2 | NM_019517 |
| 436 | scl00170439.1_29-S | ELOVL family member 6, elongation of long chain fatty acids | Elovl6 | Elovl6 | NM_130450 |
| 437 | scl42611.4_130-S | tribbles homolog 2 | Trib2 | Trib2 | NM_144551 |
| 438 | scl36717.10_331-S | RAB27A protein | Rab27a | Rab27a | NM_023635 |
| 439 | scl21321.4_587-S | coiled-coil domain containing 3 | Ccdc3 |  | XM_129987 |
| 440 | scl28794.10.1_120-S | actin, gamma 2, smooth muscle, enteric | Actg2 | Actg2 | NM_009610 |
| 441 | scl0228796.16_6-S | bactericidal/permeability-increasing protein-like 3 precursor | Bpil3 | Bpil3 | NM_199303 |
| 442 | scl23443.27.1_150-S | phospholipase C-like 4 | A930027K05Rik | Plch2 | NM_175556 |
| 443 | scl098303.1_76-S | hypothetical protein LOC98303 | D630023F18Rik | D630023F18Rik | NM_175293 |
| 444 | scl46141.4_0-S | stanniocalcin 1 | Stc1 | Stc1 | NM_009285 |
| 445 | scl016867.1_209-S | luteinizing hormone/choriogonadotropin receptor | Lhcgr | Lhcgr | NM_013582 |
| 446 | scl00319504.2_83-S | neuronal cell adhesion molecule | C130076O07Rik | Nrcam | NM_176930 |
| 447 | scl15964.10_1-S | hydroxysteroid (17-beta) dehydrogenase 7 | Hsd17b7 | Hsd17b7 | NM_010476 |
| 448 | scl22547.4.5_11-S | alcohol dehydrogenase 1 (class I) | Adh1 | Adh1 | NM_007409 |
| 449 | scl20078.15.1_71-S | hypothetical protein LOC228802 | BC018465 | BC018465 | NM_144890 |
| 450 | scl44314.9.1_18-S | aldo-keto reductase family 1, member C18 | Akr1c18 | Akr1c18 | NM_134066 |
