# Supplementary material for: Estrogen-Dependent Gene Expression in the Mouse Ovary
Source: PLoS One. 2011 Feb 9;6(2):e14672. doi: 10.1371/journal.pone.0014672 (PMC3036593; doi:10.1371/journal.pone.0014672)
Supplement: Table S2 — 78 most significant differentially genes that are most likely to represent direct biological E target genes. (0.13 MB DOC) [file pone.0014672.s002.doc]

Supplemental Table 1. 78 most significant differentially genes that are most likely to represent direct biological E target genes

|  | Name | Gene symbol | Genbank | Fold-Change |
| --- | --- | --- | --- | --- |
| 1 | carbonic anyhydrase 12 | Car12* | NM_178396 | 47.04 |
| 2 | proprotein convertase subtilisin/kexin type 6 | Pcsk6 | XM_355911 | 11.18 |
| 3 | complement component 3 | C3 | NM_009778 | 8.91 |
| 4 | complement component 4A | C4a | NM_011413 | 6.67 |
| 5 | collagen, type VIII, alpha 1 | Col8a1* | NM_007739 | 6.27 |
| 6 | glial cell derived neurotrophic factor | Gdnf | NM_010275 | 6.13 |
| 7 | protein disulfide isomerase associated 5 | Pdia5* | NM_028295 | 5.43 |
| 8 | neural cell adhesion molecule 1 | Ncam1 | NM_010875 | 4.61 |
| 9 | cell death-inducing DFFA-like effector a | Cidea* | NM_007702 | 4.56 |
| 10 | SRY-box containing gene 9 | Sox9 | NM_011448 | 4.20 |
| 11 | ankyrin 2 | Ank2* | NM_178655 | 4.08 |
| 12 | tweety homolog 1 | Ttyh1* | NM_021324 | 4.02 |
| 13 | laminin, gamma 3 | Lamc3* | NM_011836 | 3.67 |
| 14 | WD repeat domain 92 | wdr92* | NM_178909.4 | 3.53 |
| 15 | cytochrome P450, family 27, subfamily a, polypeptide 1 | Cyp27a1 | NM_024264 | 3.24 |
| 16 | dynein, axonemal, heavy chain 9 | Dnahc9* | XM_110968 | 3.13 |
| 17 | angiotensinogen | Agt | NM_007428 | 3.07 |
| 18 | a disintegrin and metallopeptidase domain 23 | Adam23 | NM_011780 | 3.01 |
| 19 | tissue inhibitor of metalloproteinase 2 | Timp2 | NM_011594 | 3.00 |
| 20 | podoplanin | Pdpn* | NM_010329 | 2.98 |
| 21 | latent transforming growth factor beta binding protein 2 | Ltbp2 | NM_013589 | 2.93 |
| 22 | glycoprotein hormones, alpha subunit | Cga | NM_009889 | 2.92 |
| 23 | tumor necrosis factor receptor superfamily, member 21 | Tnfrsf21 | NM_178589 | 2.85 |
| 24 | myosin VIIA | Myo7a* | NM_008663 | 2.85 |
| 25 | cytochrome P450, family 1, subfamily b, polypeptide 1 | Cyp1b1 | NM_009994 | 2.70 |
| 26 | platelet derived growth factor receptor, alpha polypeptide | Pdgfra | NM_011058 | 2.60 |
| 27 | dermokine | Dmkn* | NM_172899 | 2.50 |
| 28 | xanthine dehydrogenase | Xdh* | NM_011723 | 2.48 |
| 29 | hydroxysteroid 11-beta dehydrogenase 1 | Hsd11b1 | NM_008288 | 2.42 |
| 30 | laminin, alpha 2 | Lama2 | NM_008481 | 2.30 |
| 31 | laminin, beta 2 | Lamb2 | NM_008483 | 2.28 |
| 32 | claudin 10 | Cldn10* | NM_021386 | 2.25 |
| 33 | collagen, type XII, alpha 1 | Col12a1* | AK076278 | 2.23 |
| 34 | carboxypeptidase Z | Cpz* | NM_153107 | 2.23 |
| 35 | StAR-related lipid transfer (START) domain containing 13 | Stard13* | NM_146258 | 2.21 |
| 36 | angiopoietin-like 6 | Angptl6* | NM_145154 | 2.18 |
| 37 | serine (or cysteine) peptidase inhibitor, clade E, member 2 | Serpine2 | NM_009255 | 2.17 |
| 38 | endothelial differentiation, lysophosphatidic acid G-protein-coupled receptor, 7 | Edg7 | NM_022983 | 2.17 |
| 39 | angiotensin I converting enzyme (peptidyl-dipeptidase A) 1 | Ace | NM_009598 | 2.15 |
| 40 | sortilin 1 | Sort1* | NM_019972 | 2.15 |
| 41 | tensin 4 | Tns4* | NM_172564 | 2.13 |
| 42 | ST3 beta-galactoside alpha-2,3-sialyltransferase 6 | St3gal6* | NM_018784 | 2.13 |
| 43 | cDNA sequence BC034090 | BC034090* | XM_148974 | 2.11 |
| 44 | G protein-coupled receptor 125 | Gpr125 | XM_132089 | 2.08 |
| 45 | periplakin | Ppl | XM_148334 | 2.06 |
| 46 | NUAK family, SNF1-like kinase, 2 | Nuak2* | NM_028778 | 2.03 |
| 47 | B-cell leukemia/lymphoma 2 | Bcl2 | NM_177410 | 2.02 |
| 48 | discoidin domain receptor family, member 1 | Ddr1* | NM_007584 | 2.01 |
| 49 | cytochrome P450, family 51 | Cyp51 | NM_020010 | -2.05 |
| 50 | insulin receptor substrate 1 | Irs1 | NM_010570 | -2.16 |
| 51 | syndecan 1 | Sdc1 | NM_011519 | -2.18 |
| 52 | immunoglobulin superfamily, member 11 | Igsf11 | NM_170599 | -2.20 |
| 53 | scavenger receptor class B, member 1 | Scarb1 | NM_016741 | -2.24 |
| 54 | CDK5 and Abl enzyme substrate 1 | Cables1 | NM_022021 | -2.39 |
| 55 | adenosine deaminase | Ada | NM_007398 | -2.45 |
| 56 | hephaestin | Heph* | NM_010417 | -2.54 |
| 57 | sestrin 3 | Sesn3 | NM_030261 | -2.61 |
| 58 | very low density lipoprotein receptor | Vldlr | NM_013703 | -2.67 |
| 59 | secreted frizzled-related protein 4 | Sfrp4 | NM_016687 | -2.83 |
| 60 | forkhead box O1 | Foxo1 | NM_019739 | -2.87 |
| 61 | Indian hedgehog | Ihh | NM_010544 | -2.98 |
| 62 | serine (or cysteine) peptidase inhibitor, clade A, member 3G | Serpina3g* | XM_354694 | -3.01 |
| 63 | latent transforming growth factor beta binding protein 1 | Ltbp1* | NM_019919 | -3.23 |
| 64 | Gm9961 predicted gene 9961 | ENSMUSG00000055015* | AK080168 | -3.27 |
| 65 | oculocutaneous albinism II | Oca2 | NM_021879.2 | -3.27 |
| 66 | endothelin receptor type B | Ednrb | NM_007904 | -3.33 |
| 67 | multiple EGF-like-domains 10 | Megf10* | NM_001001979 | -3.41 |
| 68 | tissue inhibitor of metalloproteinase 1 | Timp1 | NM_011593 | -3.45 |
| 69 | RAS guanyl releasing protein 1 | Rasgrp1* | NM_011246 | -3.57 |
| 70 | cytochrome P450, family 17, subfamily a, polypeptide 1 | Cyp17a1* | NM_007809 | -3.89 |
| 71 | amine oxidase, copper containing 3 | Aoc3* | NM_009675 | -3.91 |
| 72 | one cut domain, family member 2 | Onecut2* | NM_194268.2 | -4.78 |
| 73 | ELOVL family member 6, elongation of long chain fatty acids | Elovl6* | NM_130450 | -5.38 |
| 74 | phospholipase C, eta 2 | Plch2* | NM_175556 | -6.94 |
| 75 | RIKEN cDNA D630023F18 gene | D630023F18Rik* | NM_175293 | -6.99 |
| 76 | stanniocalcin 1 | Stc1 | NM_009285 | -8.93 |
| 77 | hydroxysteroid (17-beta) dehydrogenase 7 | Hsd17b7 | NM_010476 | -10.83 |
| 78 | neuron-glia-CAM-related cell adhesion molecule | Nrcam* | NM_176930 | -10.16 |

Genes were ranked from most significantly up-regulated to the most significantly down-regulated according to fold change. Cut-off is ±2 fold change in expression in ArKO compared to WT (n = 3), p-value ≤ 0.05. * denote genes have not been shown to express in the ovary.
