# Supplementary figures and images for: Estrogen-Dependent Gene Expression in the Mouse Ovary
Source: PLoS One. 2011 Feb 9;6(2):e14672. doi: 10.1371/journal.pone.0014672 (PMC3036593; doi:10.1371/journal.pone.0014672)

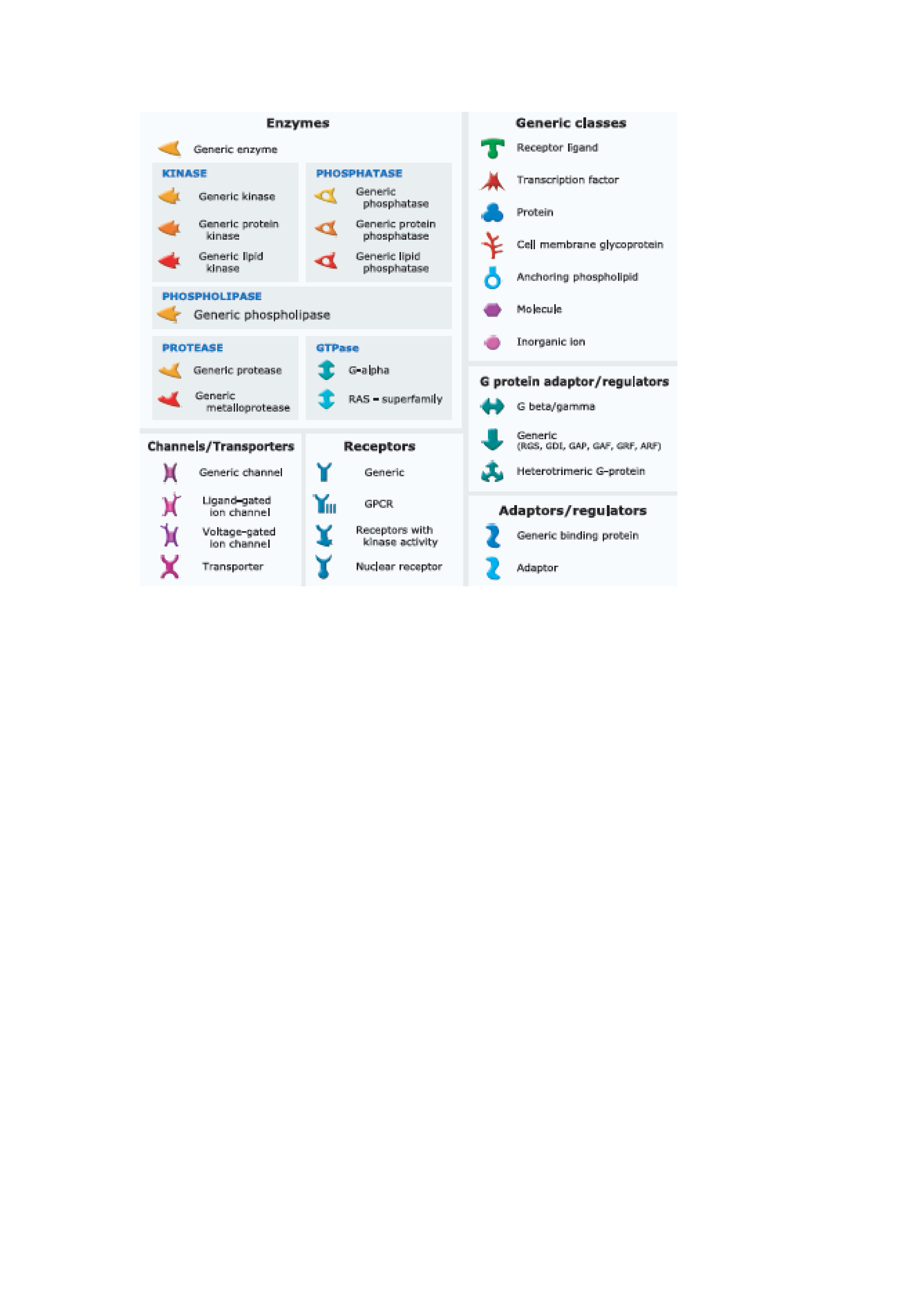

Supplement: Figure S1 — Network object legend. (3.18 MB TIF) [file pone.0014672.s003.tif]
